# Strong population differentiation in lingcod (*Ophiodon elongatus*) is driven by a small portion of the genome

**DOI:** 10.1111/eva.13037

**Published:** 2020-06-29

**Authors:** Gary C. Longo, Laurel Lam, Bonnie Basnett, Jameal Samhouri, Scott Hamilton, Kelly Andrews, Greg Williams, Giles Goetz, Michelle McClure, Krista M. Nichols

**Affiliations:** ^1^ NRC Research Associateship Program Northwest Fisheries Science Center National Marine Fisheries Service National Oceanic and Atmospheric Administration Seattle WA USA; ^2^ Pacific States Marine Fisheries Commission Under contract to Northwest Fisheries Science Center National Marine Fisheries Service National Oceanic and Atmospheric Administration Seattle WA USA; ^3^ Moss Landing Marine Laboratories Moss Landing CA USA; ^4^ Conservation Biology Division Northwest Fisheries Science Center National Marine Fisheries Service National Oceanic and Atmospheric Administration Seattle WA USA; ^5^ UWJISAO Under contract to Northwest Fisheries Science Center National Marine Fisheries Service National Oceanic and Atmospheric Administration Seattle WA USA; ^6^ Fisheries Resource Analysis and Monitoring Division Northwest Fisheries Science Center National Marine Fisheries Service National Oceanic and Atmospheric Administration Seattle WA USA; ^7^ Pacific Marine Environmental Laboratory National Oceanic and Atmospheric Administration Seattle WA USA

**Keywords:** fisheries management, heterogeneous genomic divergence, latitudinal cline, Northeast Pacific Ocean, population genomics, RAD sequencing

## Abstract

Delimiting intraspecific genetic variation in harvested species is crucial to the assessment of population status for natural resource management and conservation purposes. Here, we evaluated genetic population structure in lingcod (*Ophiodon elongatus*), a commercially and recreationally important fishery species along the west coast of North America. We used 16,749 restriction site‐associated DNA sequencing (RADseq) markers, in 611 individuals collected from across the bulk of the species range from Southeast Alaska to Baja California, Mexico. In contrast to previous population genetic work on this species, we found strong evidence for two distinct genetic clusters. These groups separated latitudinally with a break near Point Reyes off Northern California, and there was a high frequency of admixed individuals in close proximity to the break. F‐statistics corroborate this genetic break between northern and southern sampling sites, although most loci are characterized by low F_ST_ values, suggesting high gene flow throughout most of the genome. Outlier analyses identified 182 loci putatively under divergent selection, most of which mapped to a single genomic region. When individuals were grouped by cluster assignment (northern, southern, and admixed), 71 loci were fixed between the northern and southern cluster, all of which were identified in the outlier scans. All individuals identified as admixed exhibited near 50:50 assignment to northern and southern clusters and were heterozygous for most fixed loci. Alignments of RADseq loci to a draft lingcod genome assembly and three other teleost genomes with chromosome‐level assemblies suggest that outlier and fixed loci are concentrated on a single chromosome. Similar genomic patterns have been attributed to chromosomal inversions in diverse taxonomic groups. Regardless of the evolutionary mechanism, these results represent novel observations of genetic structure in lingcod and designate clear evolutionary units that could be used to inform fisheries management.

## 1 INTRODUCTION

Delineating accurate population boundaries is a critical component of harvest and protected species management; without biologically robust boundaries, estimates of population abundance, productivity, and other dynamics are very likely to be wrong. To delineate population boundaries, species assessments have typically relied on life history and demographic data (Haltuch et al., 2018). Although genetic studies provide an excellent mechanism for detecting population structure, the incorporation of genetic data into management and stock assessments has historically been slow (Hauser & Seeb, 2008; Reiss, Hoarau, Dickey‐Collas, & Wolff, 2009; Waples, Punt, & Cope, 2008). This is partly due to the difficulty commonly associated with detecting and interpreting genetic population structure in marine species with large population sizes, high dispersal potential, and few barriers to gene flow. These characteristics, which result in generally low levels of genetic differentiation, make population genetic tools better suited to define populations in an evolutionary context. In contrast, fishery managers tend to be more interested in population differences on ecologically relevant scales (Waples & Gaggiotti, 2006). Genomic techniques (i.e., approaches that generate thousands to millions of polymorphic loci) have advanced the field of population genetics with increased power for detecting population structure (Bernatchez et al., 2017; Waples & Gaggiotti, 2006) and opened the door to identifying genetic markers associated with adaptive differences (Benestan et al., 2016; Sandoval‐Castillo, Robinson, Hart, Strain, & Beheregaray, 2018). Despite these recent advances, the best available information on stock structure for most species harvested in commercial fisheries does not include insights from genomic studies (Bernatchez et al., 2017). Closing this gap in knowledge is a major need (Lynch, Methot, & Link, 2018) for species that have (a) geographic ranges that cross jurisdictional boundaries, (b) a stock status with higher uncertainty or greater management concern, and (c) ecological characteristics that may make them more likely to exhibit spatial population structure.

*Ophiodon elongatus*, Girard 1854 (lingcod) are a commercially and recreationally valuable species of groundfish, with landings and revenues within U.S. waters totaling 1,628 metric tons in 2016, and an average growth of over 200 metric tons per year since 2010 (Haltuch et al., 2018). Lingcod are distributed across international and state borders in the eastern Pacific Ocean, and as such, stocks are assessed and managed primarily along the political boundaries at the international (Canada–United States–Mexico) and national levels (Oregon–California). Boundaries also exist separating the inside waters of the Salish Sea from the outside waters of the Pacific Ocean; the Salish Sea is further divided into U.S. and Canadian waters. Within the United States, separate northern and southern lingcod stock regions, divided at the Oregon–California border, were adopted by the Pacific Fishery Management Council and federal stock assessors. This delineation was implemented due to observed differences in commercial landings data and history of exploitation, rather than reflecting any distinct breaks in lingcod genetic structure, biology, or distribution (Haltuch et al., 2018).

Lingcod exhibit characteristics that can lead to spatial population structure due to several ecological factors, including (a) reproduction is characterized by migrations to relatively shallow, coastal areas; (b) early life‐history stages inhabit nearshore and estuarine habitats; and (c) adult home ranges are relatively small with individuals generally exhibiting strong site fidelity (Hart, 1973; Jagielo, 1990; Low & Beamish, 1978; Phillips & Barraclough, 1977; Starr, O’Connell, & Ralston, 2004; Tolimieri, Andrews, Williams, Katz, & Levin, 2009). Previous population genetic studies on lingcod using allozymes, mtDNA, and microsatellites have yielded equivocal results but have primarily indicated high gene flow and a lack of population structure (Jagielo, Leclair, & Vorderstrasse, 1996; LeClair, Young, & Shaklee, 2006; Marko, Rogers‐Bennett, & Dennis, 2007). While the most recent lingcod stock assessment suggests that both northern and southern stocks have been rebuilt following an overfished state in 1999, the southern stock has shown significantly slower recovery and is designated in a precautionary zone for the purpose of U.S. federal fisheries management (Haltuch et al., 2018). These disparate population trajectories have increased scientific and management interest in developing clear insight into lingcod population structure. Using genomic analyses to identify structure could increase the accuracy of future assessments and the effectiveness of fisheries management measures, as evidenced by Andrews et al. (2018).

The objective of this study was to evaluate population structure more comprehensively using modern genomic techniques combined with high‐resolution spatial sampling throughout the bulk of the species range in order to provide updated information on population structure to aid fisheries management. To address this objective, we used restriction site‐associated DNA sequencing (RADseq) to generate thousands of polymorphic loci that sample widely throughout the genome, increasing our power to detect population structure and to identify signatures of adaptive loci. We present these results in the context of a draft lingcod genome assembly to better understand where highly differentiated RADseq loci occur in the genome.

## 2 MATERIALS AND METHODS

### 2.1 Species description

Lingcod are benthic predators endemic to the North Pacific, ranging from the Gulf of Alaska to central Baja California, Mexico (Hart, 1973). Lingcod are sexually dimorphic, with females typically growing faster and attaining larger asymptotic sizes than males. Females also reach maturity at larger sizes, between 3 and 5 years of age, whereas males are smaller but reach maturity earlier, at 2 years of age (Cass, Beamish, & McFarlane, 1990; Miller & Geibel, 1973). Additionally, lingcod exhibit a latitudinal trend in growth, longevity, and size at maturity, where individuals from northern waters generally grow faster, live longer, and mature at larger sizes than individuals from southern regions (Lam, 2019). Iteroparous spawning occurs from late winter to early spring when females move from often deeper, offshore habitat into shallow, rocky habitat where they deposit up to 500,000 eggs in high current areas (Hart, 1973; Low & Beamish, 1978). After fertilization, males will guard clutches, sometimes from multiple females, until the eggs hatch in 6–8 weeks (King & Withler, 2005; Withler et al., 2004). Lingcod have a pelagic larval duration (PLD) of around 90 days, where the first two weeks are spent in surface waters followed by 2.5 months offshore epipelagically before recruiting to a suitable habitat (Hart, 1973; Phillips & Barraclough, 1977). Young typically recruit at around 4–6 cm in length to sandy, low relief habitat adjacent to rocky, high relief substrate, which is the preferred habitat of adults (Bassett, Lindholm, Garza, Kvitek, & Wilson‐Vandenberg, 2018). Tagging studies have suggested that adults exhibit a high degree of site fidelity and a relatively small home range, although there are infrequent observations of long‐range movement in some individuals (Jagielo, 1990; Starr et al., 2004; Tolimieri et al., 2009). There is some evidence that females make longer spawning migrations than males, moving from deeper, offshore habitat to shallower spawning habitat (Low & Beamish, 1978). This is supported by the relative abundance of large females caught in the offshore trawl fishery; however, the nature and extent of this suspected spawning migration are not well characterized (Jagielo, 1990).

### 2.2 Sample collections

We chartered recreational fishing boats to collect lingcod using hook‐and‐line fishing gear on high relief rocky habitat at 25 ports from Southeast Alaska to Southern California. Sample collections out of each port typically occurred across 2–5 days. To ensure a thorough collection of lingcod across a wide range of age and size classes, both shallow (<60 m) and deep (60–170 m) nearshore and offshore reefs were targeted by our fishing efforts. Most of the individual samples analyzed in this study came from fin clips or gill tissues obtained from these collections. Supplemental tissue samples were provided by bottom fish trawl surveys conducted in lower‐relief habitat by the Alaska Fisheries Science Center's Resource Assessment and Conservation Engineering (RACE) Division and the Northwest Fisheries Science Center's Fishery Resource Analysis and Monitoring (FRAM) Division, opportunistic port sampling, and via collaborators. In total, this study included samples from 42 sites encompassing most of the species range (Table 1). All tissue samples were stored in 95% ethanol. DNA was preferentially extracted from gill tissue, which consistently yielded higher quality and quantity DNA than fin clippings (data not shown). From a single male collected in Hood Canal (Salish Sea), gill and liver tissues were flash‐frozen in liquid nitrogen for high molecular weight DNA extraction for genome sequencing; flash‐frozen tissues were stored at −80°C until DNA extraction. In our hook and line surveys, between 25 and 100 individuals were lethally sampled from each port for a related life history and demographic study (Lam, 2019); otherwise, fish were released after measurements and tissue sampling. Removal of gill tissue for sampling (~100 mg) did not appear to adversely affect individual lingcod in trials at the Seattle Aquarium. Lingcod collected via hook‐and‐line surveys were sexed externally by the presence (male) or absence (female) of a conical papilla. Additionally, we verified sex through visual inspection of gonads when lethally sampled. Lingcod were aged using the fin‐ray method (Chilton & Beamish, 1982), which has been found to have the highest accuracy, readability, and minimal between‐reader bias when compared to methods using other aging structures (Beamish & Chilton, 1977; Cass & Beamish, 1983; Claiborne, Rosenfield, Topping, Downs, & Tsou, 2016).

**Table 1:** Lingcod sampling site information for final analyses

Region	Site #	Site name	*n*	lat	long
GOA	1	South Montague	7	59.1815	−147.9763
GOA	2	East Kodiak	10	58.4054	−151.0168
GOA	3	West Chirikof	10	55.7728	−156.8757
AK	4	Nearshore Yakutat	41	59.5388	−139.9117
AK	5	ADFG stat area 375832	10	58.6167	−137.7500
AK	6	ADFG stat area 395800	16	58.3333	−139.1667
AK	7	ADFG stat area 385800	18	58.3333	−138.5833
AK	8	ADFG stat area 355702	8	57.1167	−136.0833
AK	9	Sitka	19	57.1614	−135.7697
AK	10	Beta Rock	18	55.6709	−133.7392
AK	11	Dall Island	17	55.1640	−133.3629
AK	12	Wolf Rocks	19	55.0157	−133.4981
CAN	13	Hecate Strait	7	53.6362	−131.0692
CAN	14	Queen Charlotte Sound	12	52.1842	−129.2015
CAN	15	Shellfish Section	14	48.9140	−125.4190
SS	16	San Juan Islands	11	48.4712	−123.0710
SS	17	Hood Canal	7	47.7155	−122.8790
SS	18	North Puget Sound	29	48.1415	−122.7057
SS	19	South Puget Sound	8	47.7041	−122.4821
WA	20	Cape Alava	17	48.1543	−124.7720
WA	21	Offshore La Push	16	48.0056	−125.3120
WA	22	Ocean Park	14	46.5353	−124.4240
OR	23	Cannon Beach	14	45.8400	−124.0219
OR	24	West Garibaldi	24	45.7167	−124.3524
OR	25	Stonewall Bank	27	44.5362	−124.4126
OR	26	Coos Bay	21	43.2535	−124.4637
OR	27	Mack Rock	16	42.2296	−124.4092
OR	28	Pt St George Reef	19	41.8230	−124.3624
N_CA	29	Reading Rock	9	41.3428	−124.1875
N_CA	30	Cape Mendocino	16	40.4731	−124.4932
N_CA	31	MacKerricher	9	39.4739	−123.8281
N_CA	32	Stewarts Pt	19	38.6116	−123.3797
N_CA	33	Offshore Pt Reyes	10	38.0606	−123.2999
C_CA	34	Farallon Islands	11	37.5042	−122.8582
C_CA	35	Big Sur	16	36.2082	−121.8235
C_CA	36	North Pt Conception	6	35.2333	−120.9204
S_CA	37	Santa Barbara Channel	10	34.2014	−119.6268
S_CA	38	Carrington Pt	14	34.0759	−120.0435
S_CA	39	Osbourne Bank	9	33.3636	−119.0547
S_CA	40	San Nicolas Island	18	33.3203	−119.4774
S_CA	41	San Diego	12	32.7735	−117.4687
BA	42	Colonet	3	30.7467	−116.5675

Note

General region of sampling site (*GOA,* Gulf of Alaska; *AK,* Alaska; *CAN,* Canada; *SS,* Salish Sea*; WA,* Washington; *OR,* Oregon; *N_CA,* Northern California; *C_CA,* Central California; *S_CA,* Southern California; and *BA,* Baja California, Mexico), site number (#) and name, number of samples for each site (*n*), latitude (lat) and longitude (long) of sampling sites.

### 2.3 RADseq library preparation, data filtering, and genotype calling

We extracted genomic DNA from 940 samples using the Qiagen DNAeasy Blood & Tissue 96 extraction kit (Qiagen, Inc) and then quantified extractions using a BioTek FLX800 Microplate Fluorescence Reader. Samples with extractions yielding <12.5 ng/μl were not sequenced. Individual DNA concentrations for the 841 remaining samples were normalized to 12.5 ng/μl and 125 ng of starting material was used for RADseq library prep. We used the RADseq protocol of Ali et al. (2016) to construct libraries with the following specific details: Genomic DNA from each sample was digested with the restriction enzyme *PstI*, libraries were sheared to 300–500 bp using a Qsonica sonicator (Newton, CT), and 100 bp paired‐end sequencing was conducted in 24 lanes using the HiSeq 4000 (Illumina, Inc) at the University of Oregon Genomics and Cell Characterization Core Facility (GC3F).

We used *Stacks* (Catchen, Hohenlohe, Bassham, Amores, & Cresko, 2013) to discover and identify single nucleotide polymorphisms (SNPs) from raw sequence data. Briefly, raw sequence data were quality‐filtered, demultiplexed, trimmed to 85 bp, and filtered for PCR clones using *process_radtag*s and *clone_filter* in *Stacks* v1.47, using otherwise default parameters. Subsequent data processing of individual samples was carried out in *Stacks* v2.1. In order to determine the optimal *Stacks* parameters specific to our dataset that minimized erroneous splitting or lumping of loci while simultaneously yielding a high number of polymorphic loci, we followed methods outlined in Paris, Stevens, and Catchen (2017) and Rochette and Catchen (2017) and found 3 to be the optimal value both for the maximum number of bp differences between alleles in a sample (*‐M*) and the maximum number of bp mismatches between sample loci (*‐n*). After initial trimming and filtering for low‐quality reads and PCR clones, loci were identified in each individual using *ustacks* with a minimum allele depth (*‐m*) of 3. Individual samples with fewer than 3 million reads were excluded from downstream analyses based on an exploratory examination of genotyping rates versus read depth (data not shown). A catalog of consensus loci was constructed with *cstacks* using 84 individuals from throughout the range with high coverage and *sstacks* was used to match individual sample loci to the catalog. We then used *populations* to remove loci that failed to meet the following criteria: present in ≥80% of individuals, minor allele frequency ≥1%, and maximum observed heterozygosity of 70%. We exported the resulting SNP dataset from *Stacks* and further filtered using *VCFtools* v.0.1.13 (Danecek et al., 2011). We then dropped all but the first SNP from each RADseq locus (*‐‐thin* 5000), removed loci in individuals that were below 10x depth of coverage (*‐‐minDP* 10), refiltered for loci found in ≥80% of individuals (*‐‐max‐missing* 0.8), and then removed individuals with >30% missing loci from the final dataset *(‐‐remove*), which was exported for downstream analyses. Finally, we identified and removed duplicate individuals using a custom R script written by Garrett McKinney (https://github.com/gjmckinney/IDduplicateSamples).

### 2.4 Analyses

Our analyses focused on evaluating range‐wide genetic differentiation in lingcod. It proceeded in three steps. First, we evaluated geographic patterns in genotypic differentiation using population genetic structure analyses to identify population clusters, with no a priori assumptions about population boundaries. Second, we calculated population genetic statistics for these clusters and for individual sampling site locations to quantify differentiation. Third, to better understand the underlying molecular basis and potential evolutionary mechanisms of the observed genetic differentiation, we conducted an outlier analysis, determined the genomic distribution of outlier and fixed loci using a draft lingcod genome assembly together with three other teleost genomes with chromosome‐level assemblies, and assessed mtDNA sequence diversity in a subset of individuals showing strong differentiation. These approaches are described in further detail below.

### 2.5 Population structure analyses

To identify distinct genetic clusters, we first ran population structure analyses without any a priori assumptions about population boundaries. We used a model‐based Bayesian clustering analysis implemented in *STRUCTURE* v2.3.4 (Pritchard, Stephens, & Donnelly, 2000) where ten replicates were run for each number of genetic clusters tested (K = 1–10) each with a burn‐in of 10,000 iterations and 100,000 MCMC reps with admixture allowed (NOADMIX = 0) and no prior location information (LOCPRIOR = 0). Next, we assessed the most likely number of clusters (K) across replicate runs using the Evanno method (Evanno, Regnaut, & Goudet, 2005), which assesses the rate of change in log probability of the data between successive values of K (ΔK), as implemented in *Structure Harvester* (Earl & vonHoldt, 2012). We also used the mean likelihood of the model [L(K)] for evaluating K as the Evanno method cannot detect a scenario of a = 1. Finally, *CLUMPP* v1.1.2 (Jakobsson & Rosenberg, 2007) was used to summarize results across replicate *STRUCTURE* runs and final plots were created using *DISTRUCT* v1.1 (Rosenberg, 2004). Average individual membership coefficients (Q values) to each cluster were taken from *CLUMPP* for cluster assignment. We also used principal component analysis (PCA) and discriminant analysis of principal components (DAPC), which do not rely on population genetic models, to summarize the diversity and variation across RADseq loci using the R package *adegenet* v2.1.1 (Jombart, 2008; Jombart et al., 2010). In the DAPC analysis, the optimal number of clusters (*k)* was identified with *find.clusters*, which employs a Bayesian information criterion method. Preliminary results suggested strong clustering in lingcod, which appeared to be driven by outlier loci (outlier detection analyses described below). We re‐evaluated structure patterns within initially identified clusters as well as with datasets excluding either neutral or outlier loci using *STRUCTURE* and PCAs. We also evaluated structure patterns based on sex and age when known.

### 2.6 Population genetic analyses

To evaluate genetic differentiation, we calculated population genetic statistics with individuals grouped by sampling site location (42 sites) and then by the distinct cluster assignments from *STRUCTURE* analyses for a subset of analyses including cluster pairwise F_ST_ comparisons and locus‐specific F‐statistics (excluding admixed individuals). We tested each locus over the entire dataset for being in Hardy–Weinberg proportions using an exact test based on 1,000 Monte Carlo permutations of alleles as implemented in *pegas* v0.12 (Paradis, 2010) and exact test *p* values were adjusted for multiple testing with a Benjamini–Hochberg (Benjamini & Hochberg, 1995) false discovery rate (FDR). Allele frequencies, observed heterozygosities, genetic diversity per locus and population, global and locus‐specific F‐statistics, and sampling sites pairwise F_ST_ (Weir & Cockerham, 1984) were calculated using the R package *HIERFSTAT* (Goudet, 2005). Using *boot.ppfst* and *boot.vc* in *HIERFSTAT,* we tested significance for pairwise F_ST_ comparisons and global F‐statistics, respectively, when confidence intervals of 1,000 bootstrap replicates did not overlap with zero. Mean observed heterozygosity, mean genetic diversity within populations, and genetic diversity overall were also calculated using *HIERFSTAT*. Pairwise F_ST_ estimates were subsequently recalculated following identification and removal of candidate outlier loci. Finally, we estimated contemporary effective population size (N_e_) for lingcod with individuals grouped by *STRUCTURE* identified genetic clusters using *NeEstimator* v2.1 (Do et al., 2014) under the random mating model using the linkage disequilibrium (LD) method (Waples & Do, 2008) with a MAF cutoff of 0.05 and report jackknifed 95% confidence intervals.

### 2.7 Outlier analyses

To better understand the nature of the observed genetic differentiation across sampling sites, we used two methods to detect loci putatively under divergent selection, *BayeScan* v2.1 (Foll & Gaggiotti, 2008) and the R package *pcadapt* (Luu, Bazin, & Blum, 2017). *BayeScan* uses a Bayesian regression approach to break down locus‐population F_ST_ coefficients into a population‐specific component and a global component. Loci are identified as outliers if the locus‐specific component is required to explain the observed diversity (Foll & Gaggiotti, 2008). We set prior odds to 100 with 100,000 iterations and used a false discovery rate (FDR) of 1%. *pcadapt* uses a principal component analysis to identify loci as outliers while controlling for population structure (Luu et al., 2017). Here, we identified 1 as the optimal number of principal components to consider—denoted *K*—by visualizing the proportion of variance explained by each PC after testing *K* = 1–20 and then computed *p* values for each SNP. Next, we transformed the computed *p* values into *q* values with a specified FDR of 0.05, as recommended by Luu et al., (2017), using the R package *qvalue* (Storey, Bass, Dabney, & Robinson, 2018) to identify candidate outlier loci. Loci were considered candidates for divergent selection if they were identified in both *BayeScan* and *pcadapt*.

### 2.8 Mitochondrial DNA sequencing

Because *STRUCTURE* results showed individuals strongly assigning to one of two clusters or as admixed individuals with near equal assignment to each cluster (and no observed back crossing), mitochondrial sequence divergence was evaluated a posteriori to assess the possibility of two cryptic species. Mitochondrial DNA is expected to accumulate differences relatively quickly due to a high mutation rate and smaller N_e_ than nuclear DNA, making mitochondrial markers excellent for differentiating divergent species or populations. We sequenced a 440‐bp segment of mitochondrial D‐loop using the primers H16498 5' (CCTGAAGTAGGAACCAGATG) (Meyer, Kocher, Basasibwaki, & Wilson, 1990) and Pro‐L (CTACCTCCAACTCCCAAAGC) (Crow, Powers, & Bernardi, 1997) in 45 individuals total; 15 from each of the three cluster assignment scenarios (northern, southern, and admixed). D‐loop sequences were edited and aligned using *Codon Code* software v4. We used *ape* v5.2 (Paradis & Schliep, 2019) to read alignments into R; *pegas* v0.12 (Paradis, 2010) was then used to call haplotypes and compute and construct haplotype networks. We quantified differentiation between clusters and then region sampling sites using Nei's G_ST_ (Nei & Chesser, 1983) and estimated 95% confidence intervals from 100 bootstrap replicates as implemented in *mmod* v1.3.3 (Winter, 2012).

### 2.9 Genome sequencing and assembly

High molecular weight DNA was extracted from gill tissue (preserved in ethanol) of a coastal Washington male individual using the Qiagen midi‐Prep kit (Qiagen, Inc); DNA from this individual was used for shotgun genome sequencing library prep and HiSeqX10 (150 base paired end) sequencing at the University of Washington Genome Sciences lab. High molecular weight DNA from a second individual (Hood Canal) was used for nanopore sequencing, as tissues from the first individual (stored in ethanol) did not yield the high molecular weight DNA ideal for long contiguous sequencing protocols, like that for nanopore (Oxford Nanopore Technologies, Oxford, UK). Both of these individuals were included in RADseq analyses and identified as northern cluster individuals. The Qiagen Midi‐Prep (Qiagen, Inc) and the Circulomics Nanobind Tissue Big DNA (Circulomics, Baltimore, MD) kits were both used to extract high molecular weight DNA from the second individual. Several nanopore libraries were prepared from the same individual for multiple instances of sequencing, and they included (a) three libraries prepared using the LSK‐108 library prep protocol (Oxford Nanopore Technologies, Oxford, UK) for DNA extracted with the Qiagen midi‐prep kit; two of these used the Covaris G‐tube (Woburn, MA) shearing following manufacturers protocol, and one library was prepared without shearing, (b) two libraries prepared using the Circulomics extracted DNA; one with the LSK‐109 library prep kit (Oxford Nanopore Technologies), and one using the field sequencing kit (Oxford Nanopore Technologies). All libraries were sequenced on the minION using R9.4 flow cells, and raw data were basecalled using either *Albacore* or *minKnow* (Guppy basecaller) running on minIT (all Oxford Nanopore Technologies). Genome assembly was first conducted only with the HiSeqX10 data. FastQC was used to evaluate the quality of the HiSeqX10 data, and data were then used for assembly using *MaSuRCA* v3.3.1 (Zimin et al., 2013), specifying an average insert size of 244 bases. A final genome assembly was conducted using both the HiSeqX10 and all nanopore data with *MaSuRCA* v3.3.3 with the Flye assembler (https://github.com/alekseyzimin/masurca). Assemblies were evaluated for completeness of conserved eukaryotic genes using *BUSCO* v3.1.0 with the odb9 databases (Seppey, Manni, & Zdobnov, 2019), whereby the assembly was compared to the Actinopterygii core set of genes.

Analyses of homology among nonsalmonid teleost genomes with chromosomal‐level assemblies have detected few inter‐chromosomal rearrangements; however, intra‐chromosomal reorganization is common (Pettersson et al., 2019; Rondeau et al., 2014). Here, we took advantage of high inter‐chromosomal synteny in teleost to gain insight into how contigs in our draft lingcod genome may arrange in chromosomes. This information may result in clearer insight into whether lingcod RADseq outlier loci showed genomic patterns of colocalization, a more random distribution, or some intermediate scenario. We used *raGOO* (Alonge et al., 2019), a reference‐guided contig ordering and orienting tool, to scaffold lingcod chromosome‐scale pseudomolecules (CSPs) using closely related teleosts with chromosomal‐level assemblies. We increased *‐i*, the minimum grouping confidence score for localization, from the default of 0.2 to 0.5 in order to prevent low confidence contigs from localizing to CSPs (M. Alonge, personal communication). In consideration of potential reference bias on scaffolding results, we generated three sets of lingcod CSP assemblies using the following teleost reference genomes: *Sebastes schlegelii*, Korean rockfish (CNSA accession: CNP0000222, He et al., 2019); *Gasterosteus aculeatus*, three‐spined stickleback (Dryad: https://doi.org/10.5061/dryad.h7h32, Peichel, Sullivan, Liachko, & White, 2017); and *Larimichthys crocea*, large yellow croaker (NCBI GenBank assembly accession: GCA_000972845.2, Ao et al., 2015).

### 2.10 Manhattan plots, LD heatmaps, and analysis of sex bias in markers

The RADseq contigs from the *Stacks* catalog were aligned to each of three the lingcod CSP assemblies, as well as to each reference genome, with *Bowtie2* v2.3.0 (Langmead & Salzberg, 2012) using the *‐‐very‐sensitive‐local* parameter. For loci that mapped more than once, the position with highest alignment score was kept. Finally, locus‐specific F_ST_ values generated from *HIERFSTAT* computations using 42 sampling sites were plotted to their corresponding alignment position on each lingcod CSP assembly and on each reference genome assembly using the R package *qqman* (Turner, 2018). For the lingcod CSP that contained most of the outlier loci in each of the three reference‐guided assemblies, we estimated linkage disequilibrium (LD) among all loci using r^2^ as implemented in *PLINK* v1.9 (Chang et al., 2015) and plotted LD heat maps in R. To assess whether strong selection acting on the sex‐determining chromosome could be driving the differentiation pattern, we searched for sex‐biased RADseq alleles (i.e., alleles found in significantly different proportions between males and females) and aligned the sequences to the *Sebastes‐*guided lingcod CSP assembly with the software *RADsex* (https://github.com/RomainFeron/RadSex) with the minimum coverage for a marker to be considered present in an individual (*–min_cov*) set to one. We chose to use this CSP assembly because of all the reference genomes used, it yielded the highest alignment rates of lingcod RADseq markers. We visualized *RADsex* results with the R package *radsex‐vis* (https://github.com/RomainFeron/RADSex‐vis).

## 3 RESULTS

*Stacks* filtering parameters resulted in 443,489 initial SNPs. Using *VCFtools,* we retained only the first SNP on each marker, which reduced the dataset to 228,426 SNPs. Subsequent filtering for ≥10× coverage in each locus of every individual followed by filtering for loci found ≥80% of individuals resulted in 16,749 loci. After individuals with more than 30% missing loci and duplicate individuals were removed, 611 individuals remained (Table S1).

### 3.1 Outlier analyses

We ran outlier analyses to detect loci potentially under selection and to understand how they may affect the observed genetic differentiation across sampling sites. We report these results first, as most downstream analyses relied on the identification of these loci. Out of 16,749 polymorphic SNPs, *BayeScan* and *pcadapt* identified 243 and 226 candidate outlier loci, respectively. Between detection methods, 182 outlier loci were shared and considered candidate loci under divergent selection.

### 3.2 Population structure

**Figure 1:**
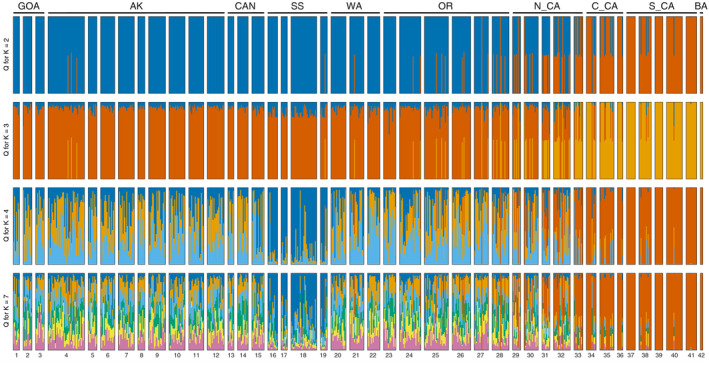
Bayesian clustering results from *STRUCTURE* for all samples and loci. Plots are shown for the four most likely *K* clusters based on (Δ*K*): *K*2 = 232.2, *K*3 = 3.4, *K*4 = 9.7, and *K*7 = 4.5. Sampling sites (numbers below each plot; see Table 1 or Table S2 for site details) are arranged from the western Gulf of Alaska following the coast east and then south down to Baja California, Mexico: *GOA* Gulf of Alaska, *AK* Alaska, *CAN* Canada, *SS* Salish Sea*, WA* Washington, *OR* Oregon, *N_CA* Northern California, *C_CA* Central California, *S_CA* Southern California, and *BA* Baja California, Mexico

**Figure 2:**
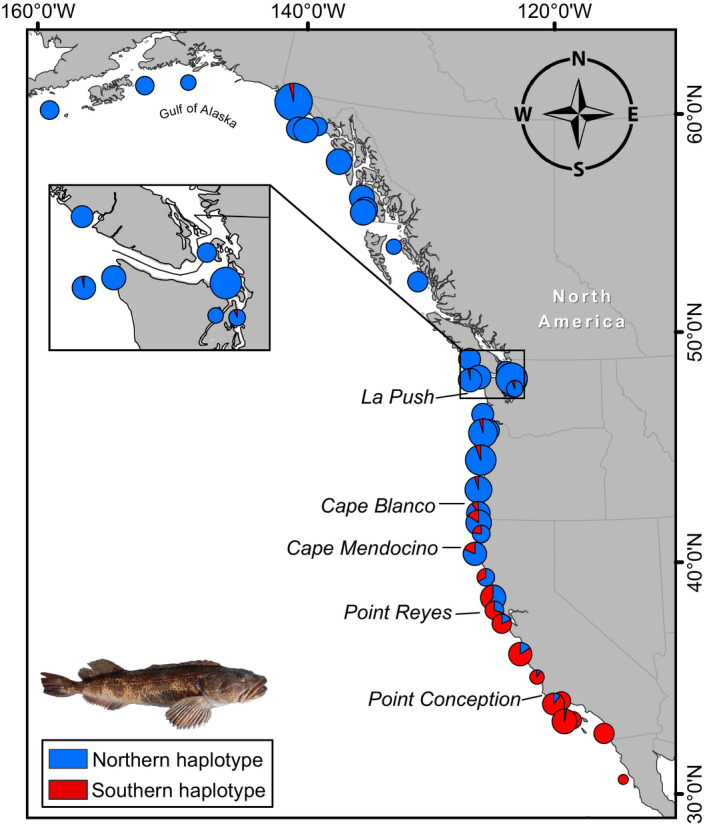
Distribution of northern (blue) and southern (red) lingcod cluster haplotype assignments based on K = 2 genetic clustering results from *STRUCTURE* analyses with all loci. Transition from predominantly northern to southern cluster occurs near latitude ~38.3°. The inset shows the inland waters of the Salish Sea

Across replicate *STRUCTURE* runs with the full dataset, (ΔK) and [L(K)] both found a = 2 was the most likely scenario with results showing two distinct genetic clusters. Individuals either definitively assigned to one of the two clusters (Q > 0.99), which segregated strongly latitudinally, or showed near equal assignment to both clusters (Q = 0.5 ± 0.05; Figure 1). As we detail below, cluster membership assignment is largely determined by nuclear haplotypes characterized by strongly linked, outlier loci; hereafter, we often refer to cluster haplotype assignments. The location at which the majority of individual memberships switch from one genetic cluster to another, or the break, appears to be centered off Northern California, where the frequency of northern cluster assignment goes from 0.605 at Stewarts Point to 0.3 offshore of Point Reyes (Figure 2; Table S2).

Individuals from the Gulf of Alaska to the northern coast of Oregon almost exclusively assign to the northern cluster haplotype except for 5 admixed individuals; three near Yakutat, AK and one individual each from south Puget Sound, WA, and off the coast of La Push, WA (Figures 1,2; Table S2). The frequency of pure northern cluster individuals drops off precipitously in Northern California with the southernmost occurrence at the Farallon Islands west of San Francisco Bay (Figure 1; Table S2). Moving south from the Farallon Islands, the frequency of admixed individuals generally decreases, while the frequency of pure southern cluster individuals increases and reaches 100% at some sites. Pure southern cluster individuals are rare north of Point Reyes, CA, with the last observation at Mack Rock, OR. Admixed individuals are the most commonly observed genotype at two sampling sites in Northern CA: MacKerricher & Stewarts Point (Figure 1; Table S2).

We repeated *STRUCTURE* analyses excluding outlier loci to determine whether these loci were responsible for the clustering we observed using the full dataset. This analysis resulted in the highest [L(K)] being K = 1—no structure—while (ΔK), which cannot detect a scenario of K = 1, suggested K = 3 was most likely (Figure S1). This result implies that nonoutlier loci are not responsible for the strong clustering observed using the full dataset. In contrast, runs containing only outlier loci yielded (ΔK) and [L(K)] values that strongly suggested K = 2 was the most likely number of genetic clusters (Figure S2), corroborating that these loci were indeed driving the pattern.

To investigate hierarchical population structure within the northern and southern clusters, we ran *STRUCTURE* analyses with only individuals belonging to each of the single clusters from the full dataset. Neither analysis suggested a scenario of K > 1 based on [L(K)]. For the northern cluster, the highest likelihood (ΔK) was K = 3; however, Salish Sea individuals showed signs of slight differentiation for all K values (Figure S3). In fact, the three *STRUCTURE* analyses that included all samples (i.e., all loci, excluding outlier loci, and exclusively based on outlier loci) showed Salish Sea individuals differentiated from other regions in most K > 2 plots (Figure 1; Figures S1,S2). These results imply that both neutral and outlier loci may differentiate Salish Sea individuals from other sites. For southern cluster individuals, ΔK suggested K = 2; however, there was no clear geographic pattern of differentiation among sampling sites or regions (Figure S4).

**Figure 3:**
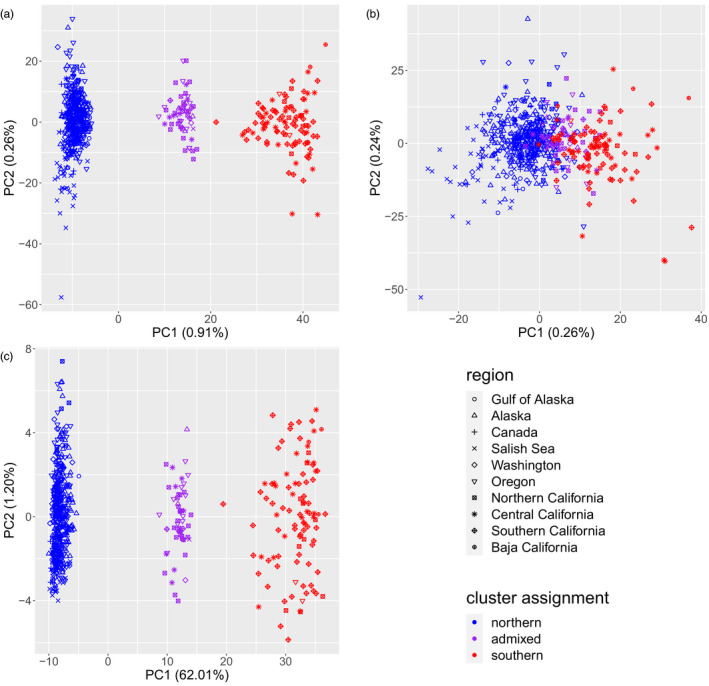
Principal components analysis for lingcod RADseq markers (a) including all loci (b) excluding outlier loci and (c) including only outlier loci with percent variation explained for PC1 and PC2. Distinct groupings seen in (a) and (c) coincide with cluster assignment results observed in *STRUCTURE* *K* = 2

In the full dataset, PCA using all loci corroborated *STRUCTURE* K = 2 clustering results by revealing three distinct groups separated along PC1 (explaining 0.91% of the variation; Figure 3a). The three groups perfectly match cluster assignment from *STRUCTURE* with northern and southern cluster individuals grouping separately and with admixed individuals falling out in the intermediate grouping. PCA without the outlier loci still shows some separation between individuals based on cluster assignment but the distinct grouping boundaries were lost (Figure 3b). A separate analysis with only the 182 outlier loci mirrored results using all loci, with PC1 explaining 61.7% of variation (Figure 3c). Within the northern and southern clusters, respectively, PCA also corroborated *STRUCTURE* analyses, where little separation was observed among regions except in the northern cluster where Salish Seas individuals showed some distinction along PC1 (Figure S5). In the DAPC analysis of population structure, (*k)* = 2 yielded the lowest BIC score (4,305.553), suggesting it was the most likely number of clusters. For this DAPC analysis, we retained 50 PCA axes and one discriminant function. Although the analysis groups admixed individuals with southern cluster individuals as opposed to northern cluster individuals, admixed individuals fall out between the northern and southern cluster groups along discriminant function 1 (Figure S6) as observed in the full dataset PCA along PC1 (Figure 3a). The population structure pattern of differentiation observed in the full dataset does not appear to be sex linked or related to age stratification, as northern, southern, and admixed individuals assigned roughly evenly to both sexes (Figure S7) and across age classes (Figure S8).

### 3.3 Population genetics

Although we detected 509 loci out of Hardy–Weinberg proportions using an FDR‐adjusted *p* value of <.05, we retained all loci for downstream analyses as these flagged markers contained most of the identified candidate outlier loci (135 out of 182). In order to estimate the strength of population differentiation, we calculated F‐statistics for different groupings of samples with and without outlier loci. F‐statistics analyses with individuals grouped by the 42 sampling sites yielded global F_ST_, F_IS_, and F_IT_ values (with 95% confidence intervals) of 0.0115 (0.0098–0.0132), 0.0024 (0.0006–0.0043), and 0.0139 (0.0112–0.0165), respectively. Locus‐specific F_ST_ varied from −0.0575 to 0.8896, but most loci exhibited low values with a small subset exhibiting higher values (Figure S9; Table S3). Significant pairwise F_ST_ comparisons ranged from 0.0006 to 0.051 with the most differentiated comparison between north Puget Sound, WA, and Colonet, Baja California (Table [Link], [Link], [Link], [Link], [Link], [Link], [Link], [Link], [Link], [Link], [Link], [Link], [Link], [Link], [Link], [Link], [Link], [Link], [Link], [Link], [Link], [Link]). Generally, pairwise F_ST_ comparisons yielded values much lower between sites on the same side of the latitudinal break (~38.3°N) and relatively high values when comparing between sites on opposite sides of the break. When candidate outlier loci were excluded, global F_ST_, F_IS_, and F_IT_ values (with 95% confidence intervals) were 0.0009 (0.0007–0.0011), 0.0021 (0.0002–0.0043), and 0.003 (0.0012–0.0052), respectively. Notably, global F_ST_ was over an order of magnitude lower, implying that most of the genome is characterized by low differentiation. Similarly, pairwise F_ST_ comparisons excluding outliers frequently showed values an order of magnitude lower between sites on the opposite side of the latitudinal break, while comparisons on the same side of the break decreased slightly, but generally remained similar to values calculated with outlier loci included (Table [Link], [Link], [Link], [Link], [Link], [Link], [Link], [Link], [Link], [Link], [Link], [Link], [Link], [Link], [Link], [Link], [Link], [Link], [Link], [Link], [Link], [Link]). However, pairwise comparisons between north Puget Sound and other northern sampling sites outside of the Salish Sea (both including and excluding candidate outlier loci) consistently produced low, but statistically significant F_ST_ values (Tables S4,S5). These results corroborate patterns of differentiation observed between Salish Sea sites and other regions in some *STRUCTURE* and PCA results (Figures 1,3; Figures S1,S2,S3,S5a).

When F‐statistics were estimated with individuals grouped by *STRUCTURE* identified clusters—excluding admixed individuals—as opposed to by sampling site, most locus‐specific F_ST_ values were again low with the same subset of loci exhibiting even higher values, suggesting strong differentiation between the two groups (Table [Link], [Link], [Link], [Link], [Link], [Link], [Link], [Link], [Link], [Link], [Link], [Link], [Link], [Link], [Link], [Link], [Link], [Link], [Link], [Link], [Link], [Link]). Indeed, 71 loci were fixed between northern and southern clusters (F_ST_ = 1) with admixed individuals being heterozygous at 69 of the 71 fixed sites (Table [Link], [Link], [Link], [Link], [Link], [Link], [Link], [Link], [Link], [Link], [Link], [Link], [Link], [Link], [Link], [Link], [Link], [Link], [Link], [Link], [Link], [Link]). All 71 fixed loci were included in the 182 outlier loci. Pairwise F_ST_ comparisons between cluster groups were orders of magnitude higher when calculated with all loci versus without outlier loci, respectively; northern and southern clusters (0.0446 & 0.0007), northern cluster and admixed individuals (0.0119 & 0.0002), and southern cluster and admixed individuals (0.0111 & 0/nonsignificant).

N_e_ estimates were infinite or undefined for the southern cluster (95% confidence interval: 959 ‐ infinite) and 29,237 (95% confidence interval: 7,643 ‐ infinite) for the northern cluster. Although high, these point estimates presumably are downwardly biased to some extent by physical linkage, which depends on the taxa‐specific number of chromosomes (Waples, Larson, & Waples, 2016). The admixed individuals likely represent an artificial population, as they appear to be heterozygous for nonrecombining haplotypes, and N_e_ estimates for this group were not considered meaningful (R. Waples, personal communication).

**Figure 4:**
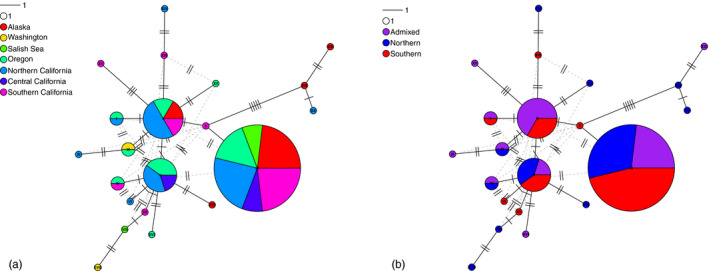
mtDNA D‐loop haplotype networks using 45 individuals (15 northern cluster, 15 admixed, and 15 southern cluster) from different regions color coded by (a) region and (b) cluster assignment. Haplotype frequency is proportional to circle size with the smallest circle representing a single representation. Number of nucleotide differences separating haplotypes is shown by perpendicular strikes through connecting line

Mitochondrial D‐loop haplotypes were shared across clusters and sampling locations (Figure 4) with no signs of haplotype fixation expected with long‐term reproductive isolation. Pairwise Nei's G_ST_ fixation indices were low between clusters (southern/northern 0.01178, southern/admixed 0.01464, and northern/admixed 0.02029), corroborating the low differentiation among cluster haplotypes visualized in the haplotype networks.

### 3.4 Genome sequencing and assembly

From the HiSeqX10 data, ~979 million reads were obtained and assembled with *MaSuRCA*. This assembly estimated the genome size of lingcod to be ~900 Mb assembled into 345,190 contigs, with a resulting contig N50 of 4.1 kbp. Using *BUSCO* to evaluate the completeness of the initial assembly against the Actinopterygii core gene set, 75% of the gene set was represented in either complete or fragmented form, and 25% of the gene set was missing. From the Nanopore data, a total of ~5.4 million reads were obtained from multiple runs (see Table [Link], [Link], [Link], [Link], [Link], [Link], [Link], [Link], [Link], [Link], [Link], [Link], [Link], [Link], [Link], [Link], [Link], [Link], [Link], [Link], [Link], [Link]). Using *MaSuRCA* for a hybrid genome assembly with the HiSeqX10 and nanopore data resulted in 2,224 contigs and 2,175 scaffolds (bridged by the 17× coverage nanopore data). The hybrid assembly had a contig N50 of 1,747 kbp. This final genome assembly, which was subsequently scaffolded with three related teleost genomes (using *raGOO*), contained 98% of the Actinopterygii conserved genes.

### 3.5 Manhattan plots, LD heat maps, and analysis of sex bias in markers

**Figure 5:**
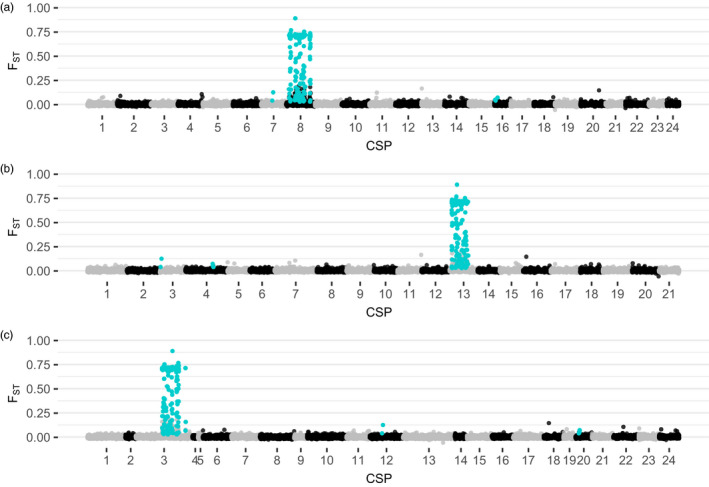
Manhattan plots aligning RADseq contigs with locus‐specific F_ST_ values based on 42 sampling sites to the draft lingcod genome assembly scaffolded into chromosomal‐scale pseudomolecules (CSPs) using the genomes of (a) *Sebastes schlegelii*, Korean rockfish; (b) *Gasterosteus aculeatus*, three‐spined stickleback; and (c) *Larimicthys crocea*, large yellow croaker, as reference guides. Outlier loci are highlighted in turquoise

To better understand the genomic distribution patterns of the outlier loci driving the strong differentiation patterns, we aligned all RADseq loci to three lingcod CSP assemblies, as well as to the respective teleost reference genomes. Alignment rates of RADseq loci to lingcod CSP assemblies and the corresponding reference genomes (i.e., percentage of loci that aligned to lingcod CSPs and reference teleost chromosomes), respectively, were 97.4% and 66.9% for the Korean rockfish, *Sebastes schlegelii*, 93.3% and 50.4% for the three‐spined stickleback, *Gasterosteus aculeatus*, and 91.7% and 50.4% for the large yellow croaker, *Larimichthys crocea*. In all three lingcod CSP assemblies, 176/182 (96.7%) outlier and 71/71 (100%) fixed loci aligned to a single CSP (Figure 5; Figure S10). Although alignment rate was expectedly lower, we observed similar patterns of outlier loci predominantly aligning to a single chromosome in the reference genomes as well (Figure S11,S12).

Linkage disequilibrium (LD) heat maps for lingcod loci on each CSP harboring the majority of outlier loci showed strong patterns of linkage in concentrated blocks corroborating outlier patterns seen in the Manhattan plots (Figure S13). Visual assessments of LD heat maps estimate the size of high LD to be ~15 Mb in length in each of the three reference‐guided lingcod CSPs, suggesting the area of localized differentiation is relatively large.

Sex‐biased marker analysis revealed a localized region on lingcod CSP23, in the *Sebastes*‐guided assembly, that exhibits a statistically significant association with sex (Figure S14). Notably, CSP8 in the *Sebastes*‐guided assembly harbors the outlier and fixed loci driving the observed differentiation pattern.

## 4 DISCUSSION

In this study, we identify two distinct genetic clusters of lingcod showing a latitudinal cline in the Eastern Pacific, with a genetic break centered off Northern California between Stewarts Point and Point Reyes. This novel delineation of population boundaries differs from previous lingcod population genetic studies, which have generally suggested coastwide panmixia (Jagielo et al., 1996; LeClair et al., 2006; Marko et al., 2007). Compared to the single mitochondrial sequence or 19–32 nuclear markers used in these prior studies, the sampling of more sites across the genome with >16,000 SNPs empowered the ability to detect this signal of population structure. Genetic clustering analyses strongly assigned individuals to either a northern or southern cluster or as admixed, with admixed individuals roughly assigned evenly to both clusters with no evidence of backcrossed individuals. Pairwise F_ST_ comparisons corroborate strong differentiation between sampling sites located on opposite sides of the latitudinal break, while low and often nonsignificant differentiation was observed when comparing sites on the same side of the latitudinal break, with the exception of the Salish Sea. There, some sites show weak, yet significant differentiation from other northern sampling sites. The strong genetic structure between northern and southern clusters is driven by outlier loci, as the signal of differentiation largely disappears in analyses excluding these loci. Alignments of RADseq loci to the three reference‐guided lingcod CSP assemblies revealed a clear pattern of outlier loci overwhelmingly aligning to a single CSP. These results point to evidence that these loci are likely colocalized on a single lingcod chromosome. A reasonable explanation for these observations given our data is a chromosomal rearrangement such as an inversion, which have recently been detected in a number of other taxa (discussed below). However, additional genome sequencing data from a southern haplotype individual is needed to confirm that this structural variation exists.

### 4.1 Strong population differentiation is driven by outlier loci

Prior genetic studies in lingcod did not detect the strong population differentiation observed in the present study. Importantly, previous studies included large sample sizes from Central California, which would almost certainly include individuals identified in this study as southern cluster or admixed; however, no striking signals of differentiation were observed along the outer coast (Jagielo et al., 1996; LeClair et al., 2006; Marko et al., 2007). These discordant results are most likely due to the differences in the molecular markers used and the extent to which these markers sampled the genome in this species. For example, Jagielo et al. (1996) and LeClair et al. (2006) used allozyme or allozyme and microsatellite markers, respectively, which sample less than a few dozen sites in the nuclear genome, while Marko et al. (2007) specifically evaluated population structure using cytochrome oxidase I in the mitochondrial genome. Sampling thousands of markers distributed widely throughout the genome allowed us to detect a very strong signal of divergence driven by a small number of outlier loci likely colocalized to a single region in the genome. In fact, when we excluded outlier loci from analyses our results corroborate previous findings of weak genetic structure, as does our mtDNA analysis using D‐loop, which showed no obvious haplotype lineage sorting based on cluster assignment or sampling region. Although males exhibit territorial behavior as nest guarders and are generally thought to move less than females (Stahl, Green, & Vaughn, 2014), we saw no evidence for bias in the distribution of sexes among genetic clusters (Figure S7); moreover, the putative sex CSP is different than the CSP harboring all of the outlier loci (Figure 5; Figure S14). Similarly, we did not detect bias in these results among age classes (Figure S8). These findings suggest cluster assignment is not sex dependent nor associated with a recruitment anomaly.

With the exception of Salish Sea sites, genetic clustering analyses within northern and southern clusters lack patterns of differentiation. Although Jagielo et al. (1996) and Marko et al. (2007) did not detect strong structure, both suggested weak genetic differentiation between the inside waters of the Salish Sea and the outer coast. However, LeClair et al. (2006) failed to find the same genetic signal with additional individuals from the Salish Sea and the increased power of microsatellite markers in conjunction with allozyme data from Jagielo et al. (1996). Although we observed major structure associated with outlier loci, we also detect weak signals of differentiation and substructure between lingcod in the Salish Sea and the Pacific Ocean both with and without outlier loci. Marko et al. (2007) estimated asymmetrical migration rates in this region with net emigration from the inner waters of the Salish Sea to the outer coast, which coincides with surface‐water circulation patterns (Ebbesmeyer et al., 1984) and larval life‐history behavior—lingcod larvae remain in surface waters for the first two weeks of their 90‐day PLD (Phillips & Barraclough, 1977). With reduced connectivity due to the net outflow of surface water and migrants, the inland waters of the Salish Sea may rely on self‐replenishment as suggested by Marko et al. (2007). That hypothesis is supported by our results, which show weak but significant population differentiation between the Salish Sea and outer coast.

### 4.2 Possible mechanisms for differentiation

There are a number of evolutionary mechanisms that can lead to the strong differentiation observed between northern and southern cluster lingcod; here, we evaluate several possibilities before discussing evidence for a chromosomal inversion. It is conceivable that the northern and southern clusters represented cryptic species interbreeding and producing sterile F1 hybrids (admixed individuals); however, the lack of divergence in mtDNA and the heterogeneous genomic patterns do not support this alternate hypothesis. If pre‐ or postzygotic barriers prevented backcrossing of admixed individuals with pure cluster individuals, we would expect no gene flow between clusters and therefore significant differentiation of mitochondrial haplotypes as well as strong patterns of differentiation throughout the entirety of the nuclear genome. Yet, we saw no evidence of D‐loop haplotype segregation in northern or southern clusters (Figure 4b) corroborating the high levels of gene flow in the mitochondrial genome reported by Marko et al. (2007). Although patterns of mitochondrial and nuclear discordance have been observed between sister rockfish (*Sebastes*) species, a high proportion of haplotypes were found to segregate in these cases (Burford & Bernardi, 2008; Kai, Park, & Nakabo, 2012; Muto, Kai, & Nakabo, 2011; Narum, Buonaccorsi, Kimbrell, & Vetter, 2004). Additionally, these studies detected strong nuclear divergence using amplified fragment length polymorphism (AFLP) and microsatellite markers, which sample orders of magnitude fewer regions of the genome, suggesting the signal of nuclear divergence was widespread.

Another alternative hypothesis for the differentiation pattern we observed in lingcod is divergent selection in the absence of a chromosomal rearrangement, which is expected to produce a distinctive region of elevated divergence or “genomic islands of divergence.” However, islands derived from divergent selection are expected to be dispersed throughout the genome and on the order of kilobases not megabases (Kim & Stephan, 2002; Lamichhaney & Andersson, 2019; Nosil, Funk, & Ortiz‐Barrientos, 2009). This scenario appears unlikely in lingcod where we consistently observed the colocalization of outlier and fixed loci to a region around 15 megabases in size on a single lingcod CSP across reference‐guided assemblies (Figure 5; Figure S10).

Yet another possibility is that the observed clusters represent recently isolated populations characterized by strong sexual selection on the sex‐determining chromosome (Martin et al., 2013; Nosil et al., 2009; Qvarnström & Bailey, 2009; Ravinet et al., 2017; Sæther et al., 2007), but this hypothesis is inconsistent with our findings as the outlier loci driving the strong differentiation patterns localize on CSP8 in the *Sebastes*‐guided assembly (Figure 5a; Figure S10a) while the sex‐determining markers localize on CSP23 (Figure S14). Interestingly, this result corroborates previous work on sex differentiation in lingcod that suggested males are the heterogametic sex (Rondeau, Laurie, Johnson, & Koop, 2016).

Chromosomal inversions, which have been detected at an increasing rate due to the proliferation of genomewide sequencing techniques, have been implicated in a number of recent studies evaluating population differentiation and the genetic basis of life histories (Berg et al., 2016; Knief et al., 2016; Lundberg et al., 2017; Sodeland et al., 2016; Wellenreuther & Bernatchez, 2018). Here in lingcod, we observed similar patterns detected across inversion studies: Highly localized genomic regions explain most observed variation, where individuals have strong affinities to discrete genetic groups. For example, the PCA pattern we observed in lingcod (Figure 3) has been seen among migratory and nonmigratory Atlantic cod, *Gadus morhua*, (Barth et al., 2019; Berg et al., 2016), Australian zebra finch, *Taeniopygia guttata*, (Knief et al., 2016), and the willow warbler, *Phylloscopus trochilus*, (Lundberg et al., 2017), where homokaryotypes for inverted and noninverted haplotypes, respectively, grouped separately along a PC axis with heterokaryotypes (individuals heterozygous for the inverted and noninverted region) clustering between the respective homokaryotype groups. Lundberg et al. (2017) failed to differentiate among warblers with different inversion haplotypes when loci located on inversion blocks were removed. In lingcod, we observed partial overlap among individuals from separate clusters (i.e., northern, southern, and admixed) when we removed outliers, although some differentiation was still observed (Figure 3b). The distinct *STRUCTURE* pattern we observed for K = 2 for all loci and outlier loci only analyses is also consistent with results seen in known inversion systems such as the Atlantic cod, where heterokaryotypic individuals assigned roughly equally to clusters homozygous for inverted and noninverted haplotypes, respectively (Kess et al., 2019). These patterns can result from systems where the colinear regions of the genome are generally characterized by high levels of gene flow, and consequentially low divergence and inversion haplotypes may be the genomic region driving differentiation patterns among individuals.

Genomic island of divergence can also be produced through localized recombination suppression between inverted and noninverted regions in the presence of otherwise unimpeded gene flow throughout the colinear regions (Nosil et al., 2009). We consistently observed a pattern of outlier loci colocalizing to a single genome region when lingcod RADseq markers were aligned to three reference‐guided lingcod CSP assemblies. The pattern was also seen when aligning loci to the chromosome‐level reference genome assemblies, which includes species that span three teleost orders. Additionally, the lingcod outlier markers show strong linkage disequilibrium (LD), a pattern also seen in diverse taxa where inversions have been found (Joron et al., 2011; Knief et al., 2016; Sinclair‐Waters et al., 2018). Inter‐chromosomal synteny appears to be well conserved in divergent teleosts lacking recent whole‐genome duplications (Pettersson et al., 2019; Rondeau et al., 2014). Indeed, the fact that lingcod outlier loci overwhelmingly map to a single lingcod CSP and to a single chromosome in all three reference teleost genomes suggests a high level of chromosomal synteny and some conservation of intra‐chromosomal syntenic blocks. We interpret this consistent pattern as evidence that genetic differentiation in lingcod is attributed to loci colocalized to a region, likely characterized by an inversion, on a single chromosome. Future work to construct a southern cluster derived genome assembly will be important to properly characterize the size, nature, and location of the putative inversion harboring these loci promoting genetic differentiation between the northern and southern genetic groups.

### 4.3 Potential factors driving divergence and latitudinal cline

Lingcod exhibit a very clear latitudinal cline with respect to genetic cluster assignment (Figure 2). Endler (1977) postulates that clines can arise from one or more of four scenarios; random genetic drift, secondary contact between previously isolated populations, steep or discontinuous differences in environmental conditions, and continuous change in the environment. Although these scenarios are not mutually exclusive, the evidence suggests that the distinct genetic cline in lingcod is most likely a product of selection driven by change (continuous and/or discontinuous) in environmental conditions. It is possible that genetic drift may have contributed to the establishment of the cline in the presence of a historical physical barrier; however, the cline appears to be maintained by selection. For one, outlier loci are almost entirely found on a single CSP. If drift were responsible for the clinal distribution of haplotype clusters, one would also expect other genomic regions to show differentiation patterns due to drift. However, most of the genome outside the region of differentiation shows signs of high gene flow, including in the mitochondrial genome. Although the realized larval dispersal may be less extensive than the potential dispersal potential (Lester & Ruttenberg, 2005; Lester, Ruttenberg, Gaines, & Kinlan, 2007), even low levels of gene flow, in the absence of selection, would rapidly homogenize cluster haplotype distributions and quickly erode the latitudinal cline (Slatkin, 1987). Secondary contact between previously isolated populations also is not supported; our lingcod D‐loop haplotype networks based on cluster assignment showed no evidence of fixation or segregation nor do previous lingcod mitochondrial haplotype analyses (Marko et al., 2007). However, the pattern we observe in lingcod does suggest that cluster haplotypes, which are defined by the colocalized outlier loci, may carry a fitness component with respect to latitude. That is, northern cluster haplotypes appear to be advantageous in the northern portion of the range while southern cluster haplotypes appear to carry an advantage in the southern portion of the range. The California coastline, where we see the steepest change in frequency of cluster assignment, has also produced selection‐driven population genetic breaks in marine invertebrates (Kelly & Palumbi, 2010; Sotka, Wares, Barth, Grosberg, & Palumbi, 2004) and fish (Sivasundar & Palumbi, 2010). In these examples, the selective pressures driving the patterns are unknown but the authors point out well‐known differences in abiotic factors, such as sea surface temperature and upwelling (Checkley & Barth, 2009; Huyer, 1983; Magnell et al., 1990), and note that Central California represents a broad transition zone between northern and southern taxa with many species range limits found in the area (Allen, Horn, & Pondella, 2006). However, the effects of lingcod cluster haplotypes on fitness with respect to environment need to be formally tested and the presence of a chromosomal inversion needs to be demonstrated in future work.

### 4.4 Fishery management implications

Our study sampled lingcod throughout their geographic range (with a limited sample from the waters of Mexico) and found evidence for two distinct genetic clusters with the break centered between Stewarts Point and Point Reyes, California. These results emphasize the need to consider transboundary stock assessments and to reconsider the location of the current stock assessment and management boundary located at the U.S. Oregon–California border (Haltuch et al., 2018). Although most of the genome is characterized by low differentiation, suggesting significant levels of connectivity, the putative chromosomal inversion haplotypes are on different demographic trajectories and meet the criteria for distinct and independent stocks (Waples, 1998). Specifically, the putative inversion haplotypes are genetically isolated from each other and the strong latitudinal cline suggests they may be associated with adaptive differentiation. In managed species characterized by inversions, such as Atlantic cod (Kirubakaran et al., 2016), optimal strategies will aim to maintain haplotype polymorphisms that confer adaptive advantages (Bernatchez et al., 2017; Funk, McKay, Hohenlohe, & Allendorf, 2012). Although the adaptive nature of the putative lingcod inversion is unknown, there is more risk in managing discrete stocks as a single population as opposed to managing a panmictic population as distinct populations (Hauser, Adcock, Smith, Ramírez, & Carvalho, 2002; Kerr et al., 2017; Laikre, Palm, & Ryman, 2005; Okamoto et al., 2019; Spies & Punt, 2015; Sterner, 2007).

The southern lingcod stock (California), as it is currently assessed, has experienced slower recovery and greater fishing pressure than the northern stock (Haltuch et al., 2018). The southern stock consists of individuals from both the northern and southern genetic cluster, but is dominated by the southern cluster south of the Farallon Islands (Figure 2; Table S2). Estimates of effective population size (*N_e_*) are large for both clusters, indicating population sizes are large or were in the recent past. Although replenishment via dispersal of northern cluster larvae to Southern California seems highly plausible based on a 90‐day PLD, the latitudinal cline suggests that the realized dispersal may be less extensive than potential dispersal (Lester & Ruttenberg, 2005; Lester et al., 2007), and/or the northern cluster individuals have lower fitness in Southern California. Signals of high gene flow in regions of the genome outside the putative inversion, excluding Salish Sea sites, and the steepness of the latitudinal cline suggest the latter plays a larger role. Regardless, it appears that Southern California lingcod predominantly rely on recruitment of the southern cluster haplotype. A management strategy that maintains southern cluster recruitment will likely be important for continued southern stock recovery. Within the northern cluster, the Salish Sea shows signs of weak but significant differentiation from outer coast sites suggesting relatively restricted connectivity. These results suggest that prudence should be taken in changing the current management boundaries separating the Salish Sea from the outer coast. More work is needed to elucidate how management of Salish Sea lingcod as a separate population could influence long‐term sustainability. Taken together, international, national, and state management agencies may want to consider stock productivity and assessment modeling in the context of these genetic and other ecological factors that may contribute to differences in their productivity and survival.

Finally, a key quandary in drawing management boundaries for lingcod is the consideration of individuals that are admixed between the northern and southern genetic groups, and the historical and future location of these boundaries with changing ocean conditions. Our working hypothesis is that these individuals are heterokaryotypic for an inversion on a single chromosome, and the fitness consequences for these individuals are uncertain. In lingcod, positive identification of the inversion and understanding the role putative heterokaryotypes play is an important future line of investigation. Specifically, understanding their influence on productivity and connectivity of the northern and southern clusters will reduce uncertainty in future assessment outputs. How cluster haplotypes may affect observed regional variation in growth rates, size at maturity, longevity, and mortality rates (Lam, 2019) is currently being investigated.

## 5 SUMMARY

Across geopolitical boundaries of the eastern Pacific Ocean, we identified two notably distinctive genetic clusters of lingcod, with a strong genetic break centered off Northern California (~38.3°N latitude). This genetic break is driven entirely by a small number of loci in linkage disequilibrium that are likely colocalized on a single chromosome that may be characterized by an inversion. Future work is necessary to confirm the presence and nature of the putative inversion; if confirmed, additional work will need to evaluate the ecological life‐history significance and fitness implications of such an inversion. We also found evidence for substructure within the northern cluster based on weak but significant differentiation between the inland waters of the Salish Sea and the outer coast that is likely driven by reduced connectivity between these areas. Current stock boundaries for management of the species differ somewhat from the patterns we observed. Our results imply that for lingcod, as with many species, an evaluation of how genetic population structure, jurisdictional boundaries, and life‐history considerations come together to influence assessment accuracy is likely to provide important insights for management of the species.

## CONFLICT OF INTEREST

None declared.

## Supporting information

Fig S1

Fig S2

Fig S3

Fig S4

Fig S5

Fig S6

Fig S7

Fig S8

Fig S9

Fig S10

Fig S11

Fig S12

Fig S13

Fig S14

Table S1

Table S2

Table S3

Table S14

Table S15

Table S16

Table S17

Table S18

## Data Availability

All sequences are openly available and deposited at NCBI. Demultiplexed, quality‐filtered forward and reverse DNA sequence reads for lingcod individuals sequenced in this study, and the draft lingcod assembly are available at the sequence read archive (SRA) under the BioProject PRJNA595583 http://www.ncbi.nlm.nih.gov/bioproject/PRJNA595583. The mtDNA sequences can be found on GenBank with accession numbers MT498360‐MT498404.
