# Supplementary figures and images for: Strong population differentiation in lingcod (Ophiodon elongatus) is driven by a small portion of the genome
Source: Evol Appl. 2020 Jun 29;13(10):2536–54. doi: 10.1111/eva.13037 (PMC7691466; doi:10.1111/eva.13037)

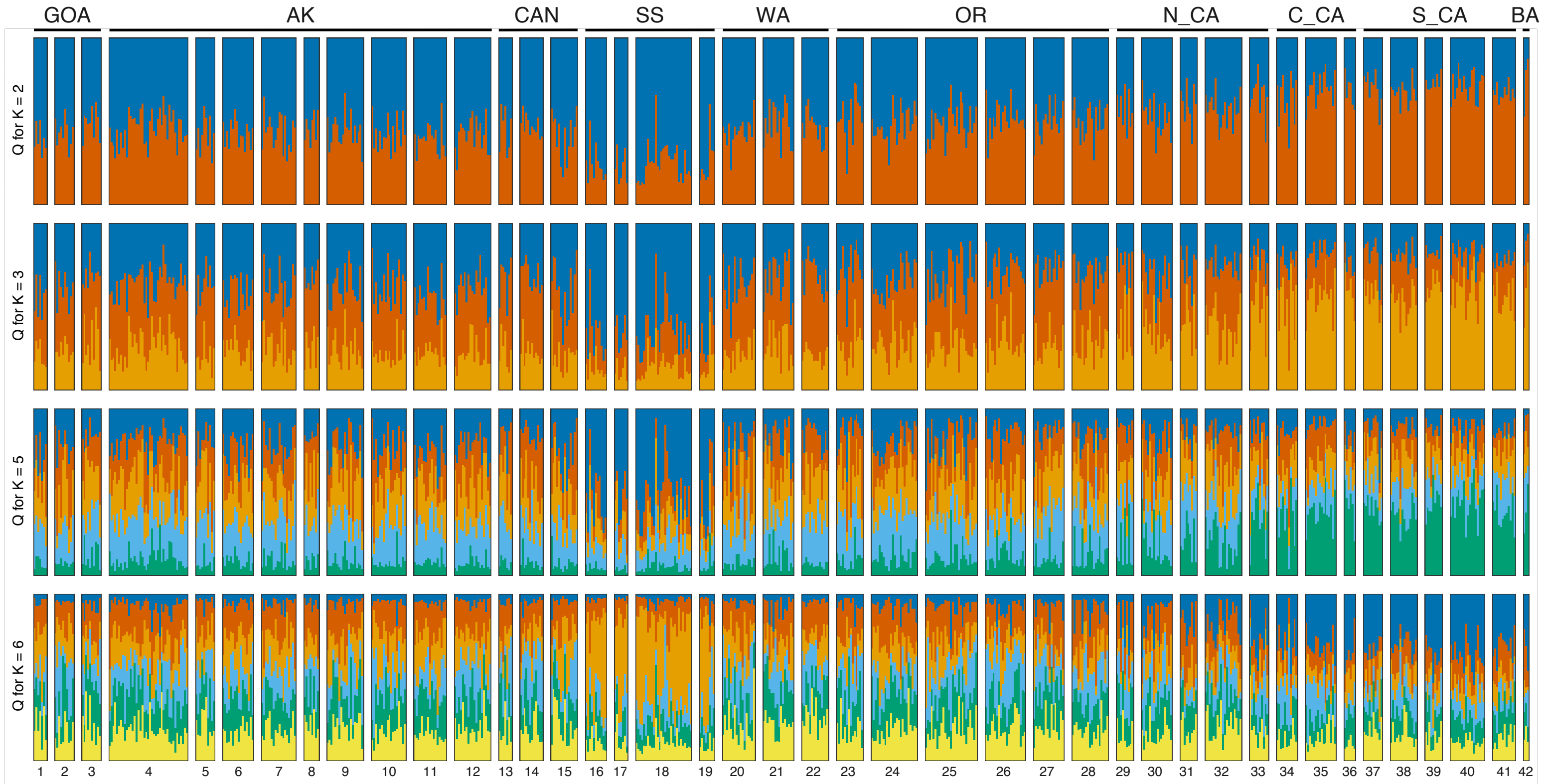

Supplement: Supplementary file 1 — Fig S1 [file EVA-13-2536-s001.pdf]

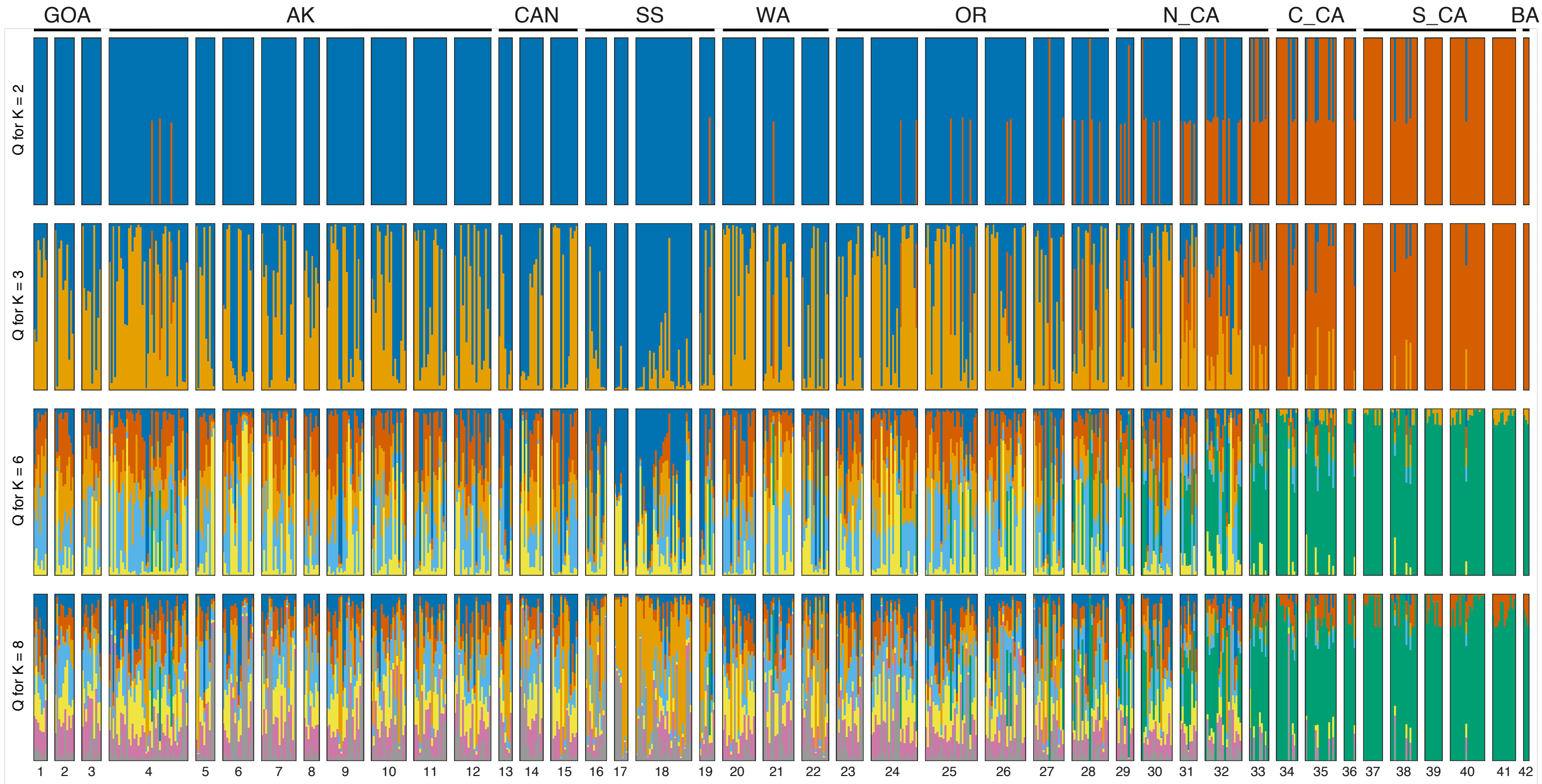

Supplement: Supplementary file 2 — Fig S2 [file EVA-13-2536-s002.pdf]

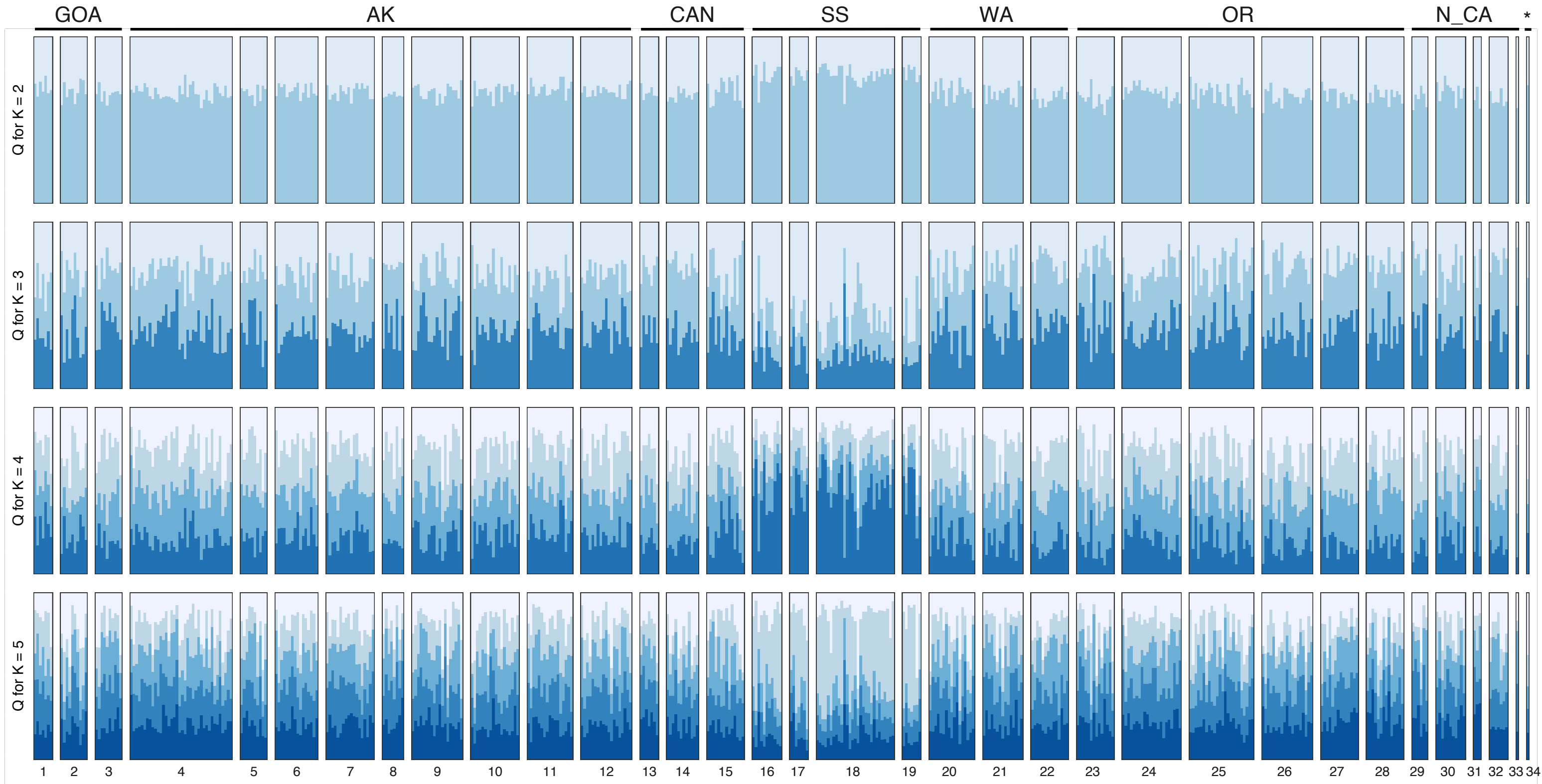

Supplement: Supplementary file 3 — Fig S3 [file EVA-13-2536-s003.pdf]

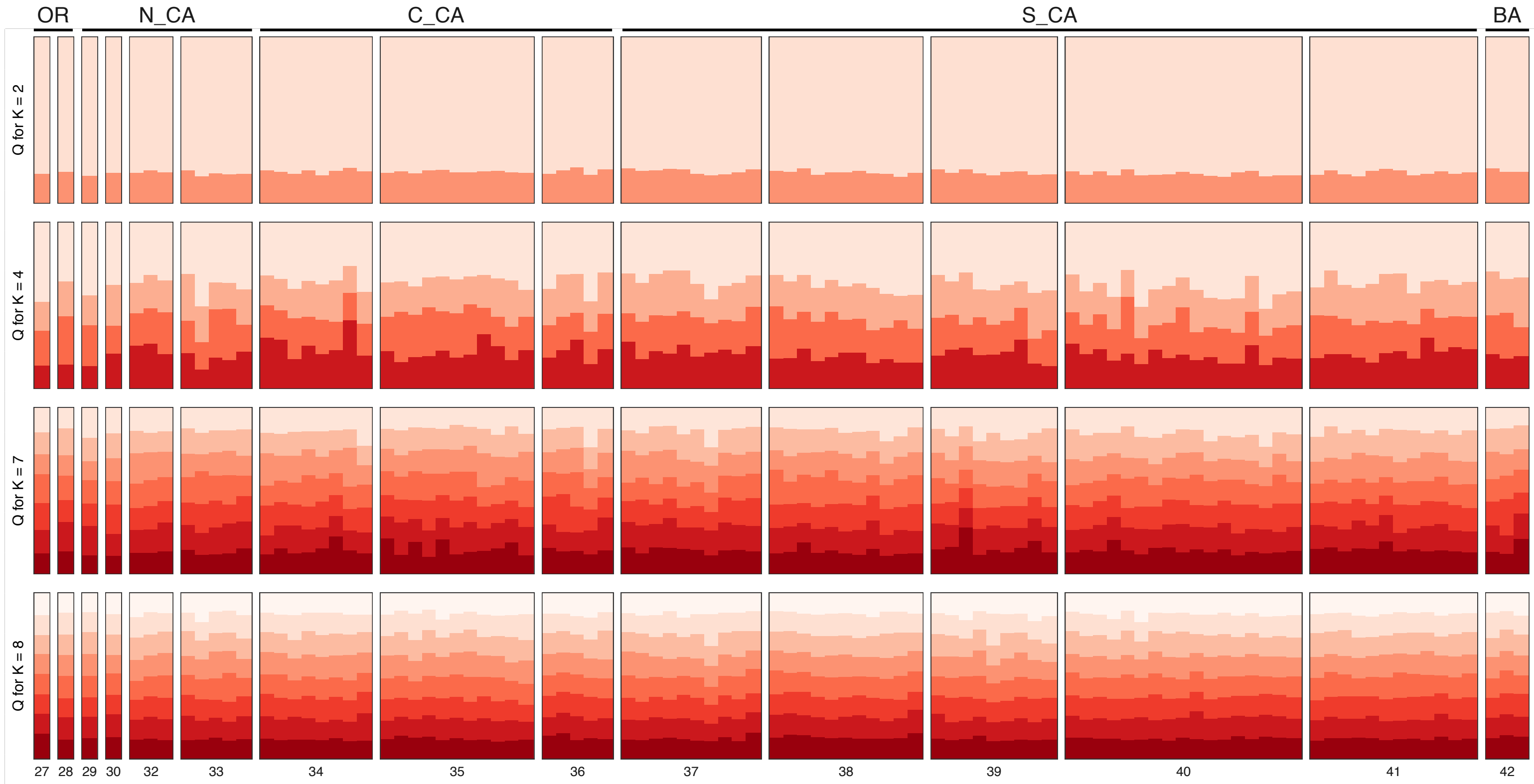

Supplement: Supplementary file 4 — Fig S4 [file EVA-13-2536-s004.pdf]

**A**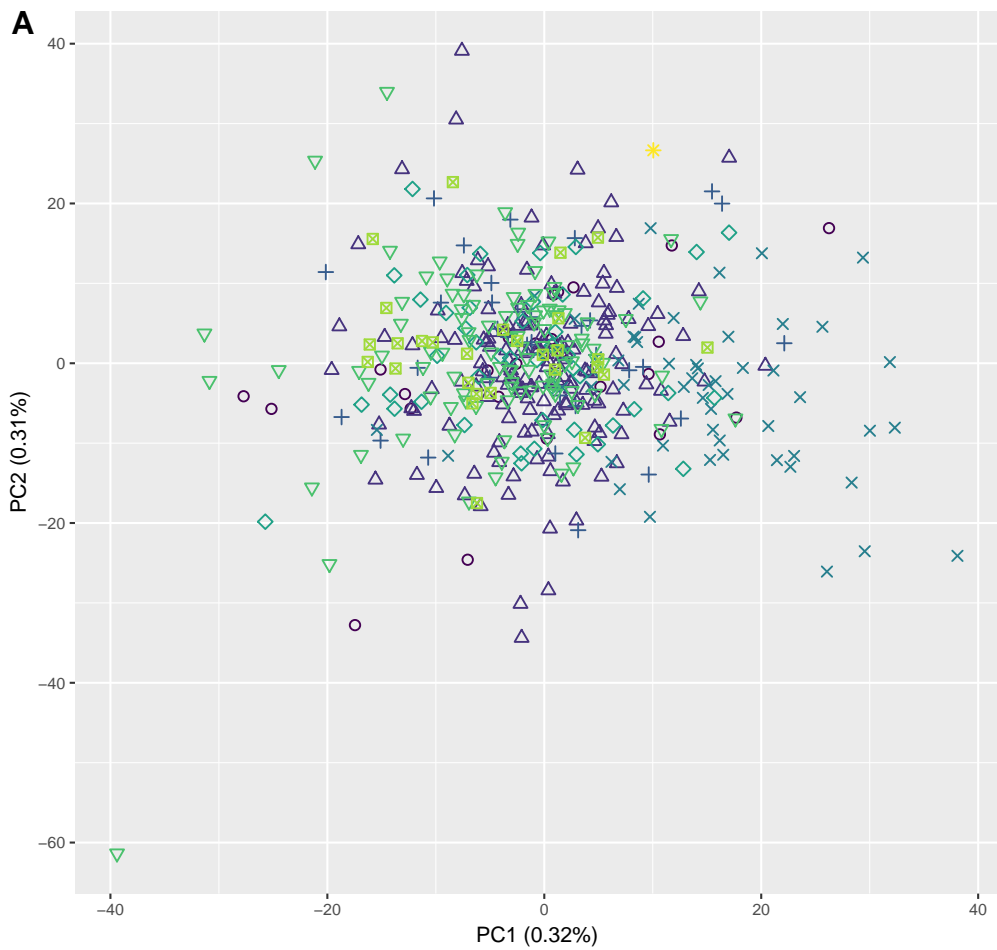**B**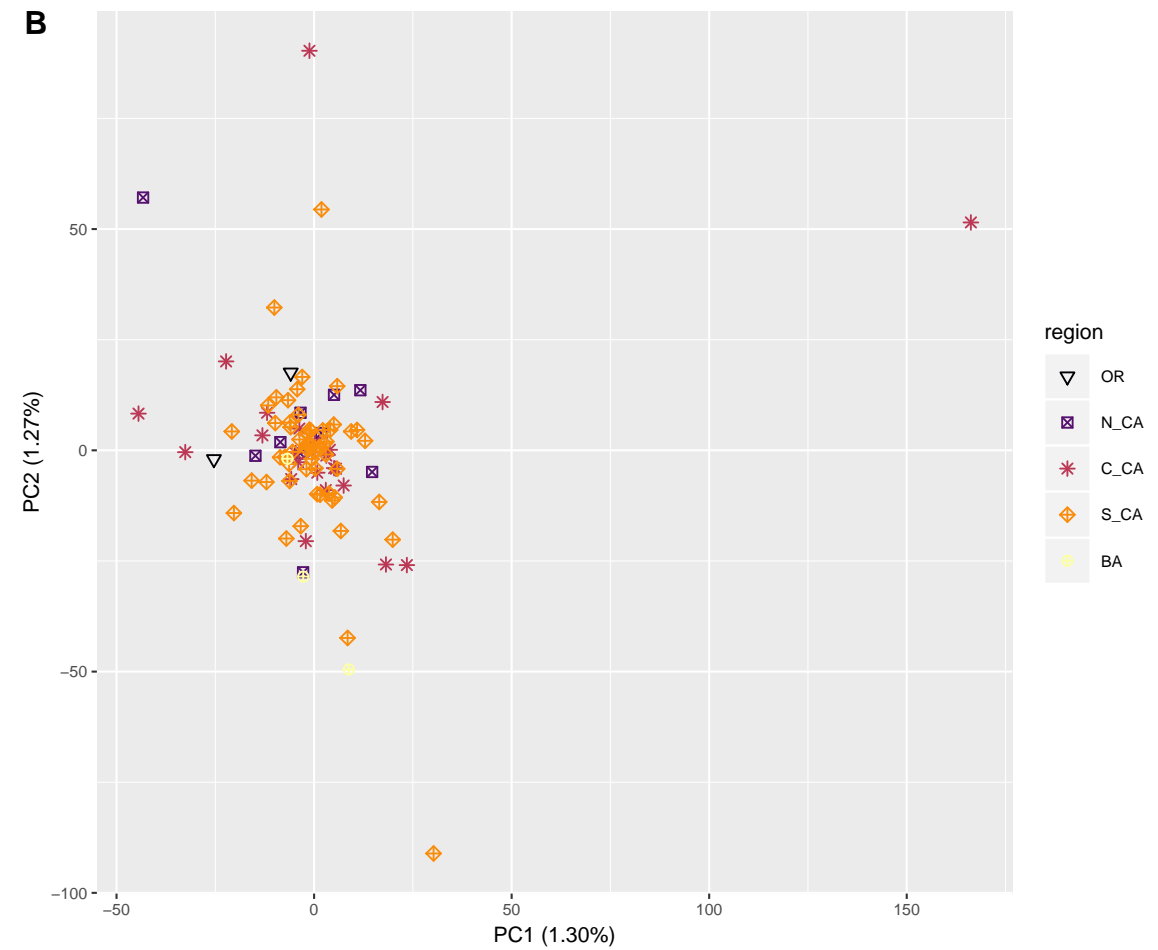

Supplement: Supplementary file 5 — Fig S5 [file EVA-13-2536-s005.PDF]

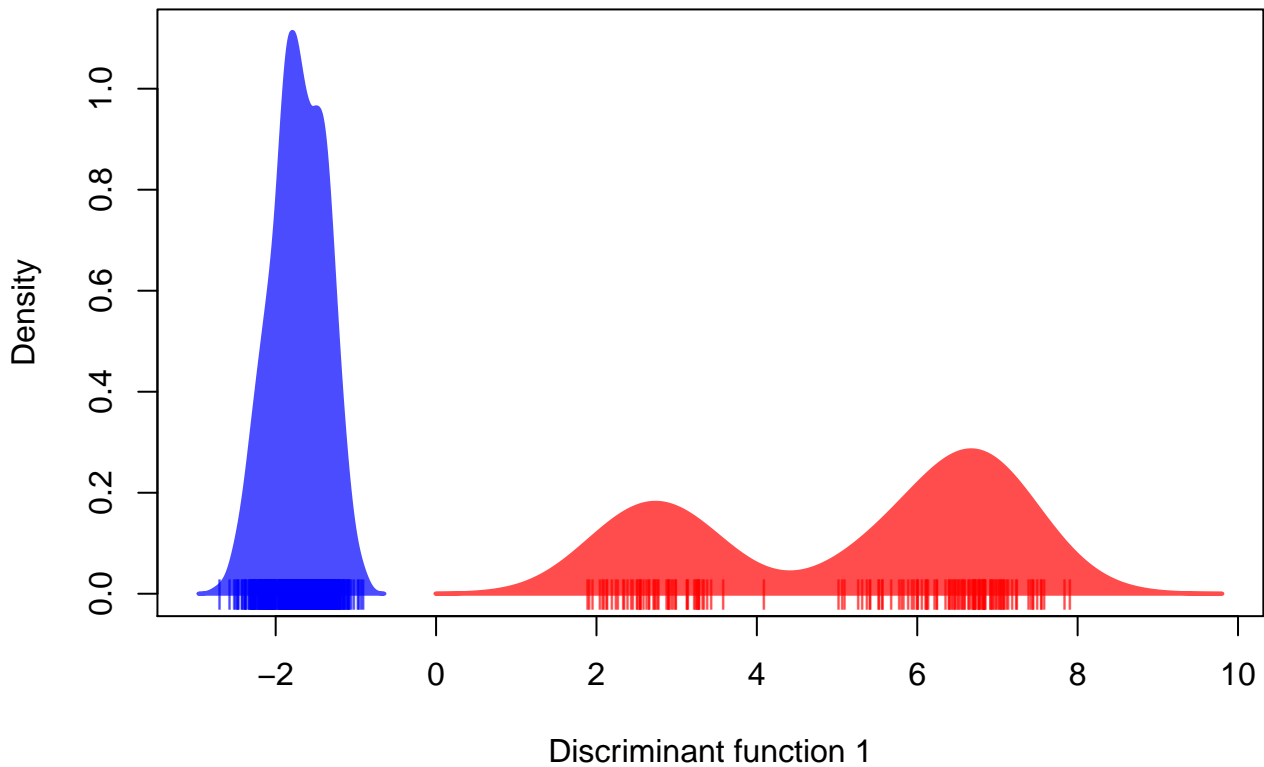

Supplement: Supplementary file 6 — Fig S6 [file EVA-13-2536-s006.pdf]

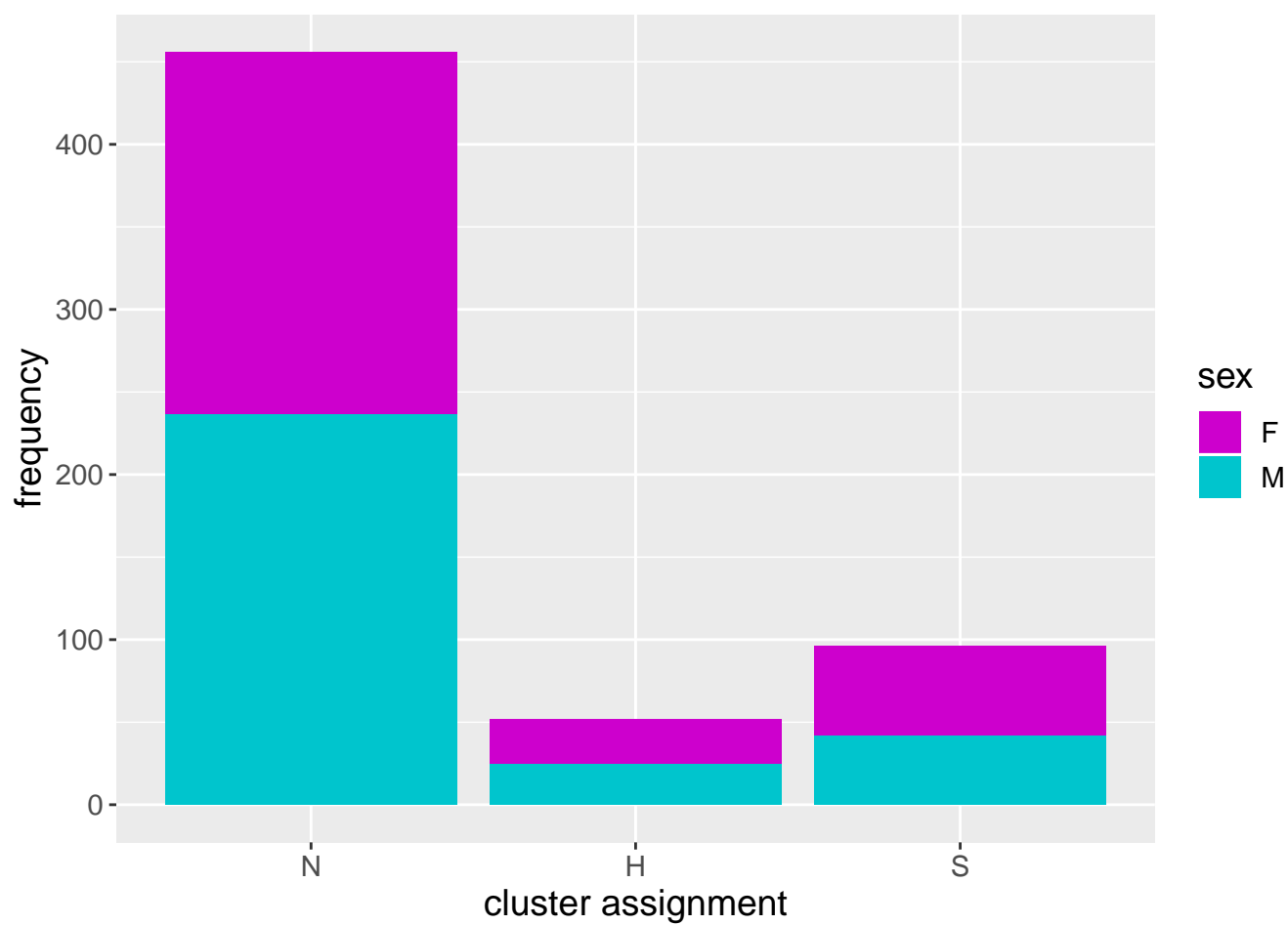

Supplement: Supplementary file 7 — Fig S7 [file EVA-13-2536-s007.pdf]

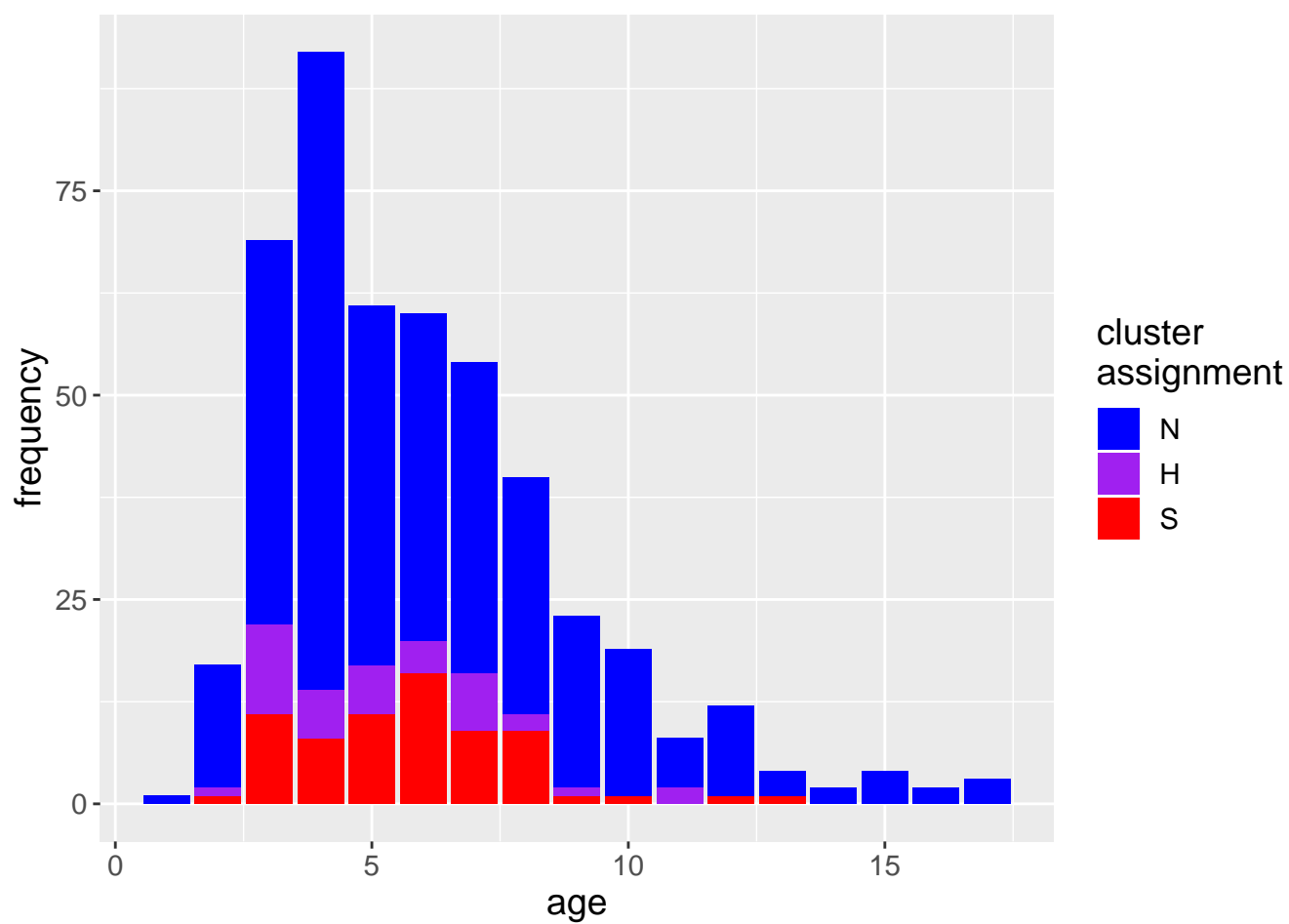

Supplement: Supplementary file 8 — Fig S8 [file EVA-13-2536-s008.pdf]

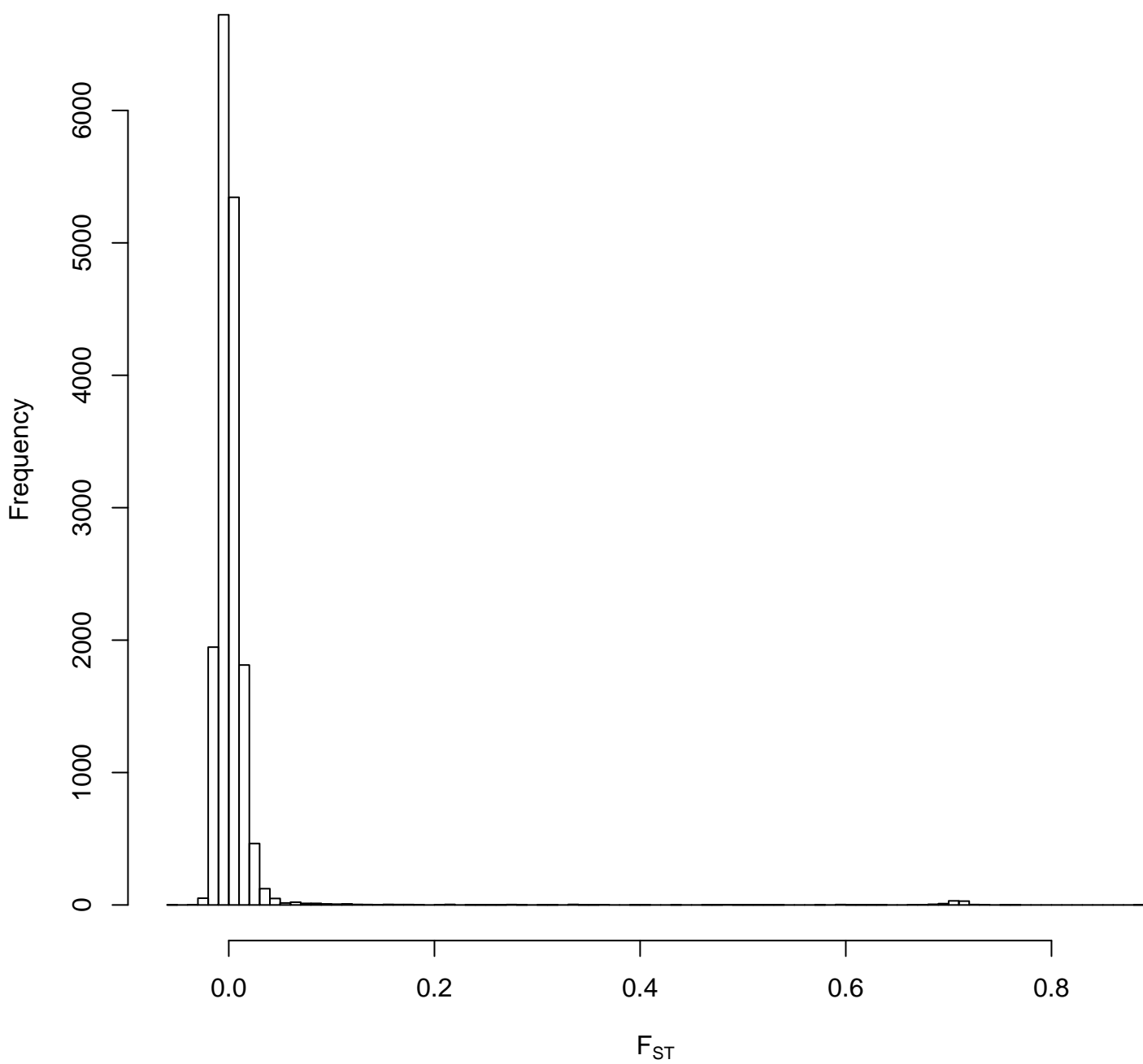

Supplement: Supplementary file 9 — Fig S9 [file EVA-13-2536-s009.pdf]

**A**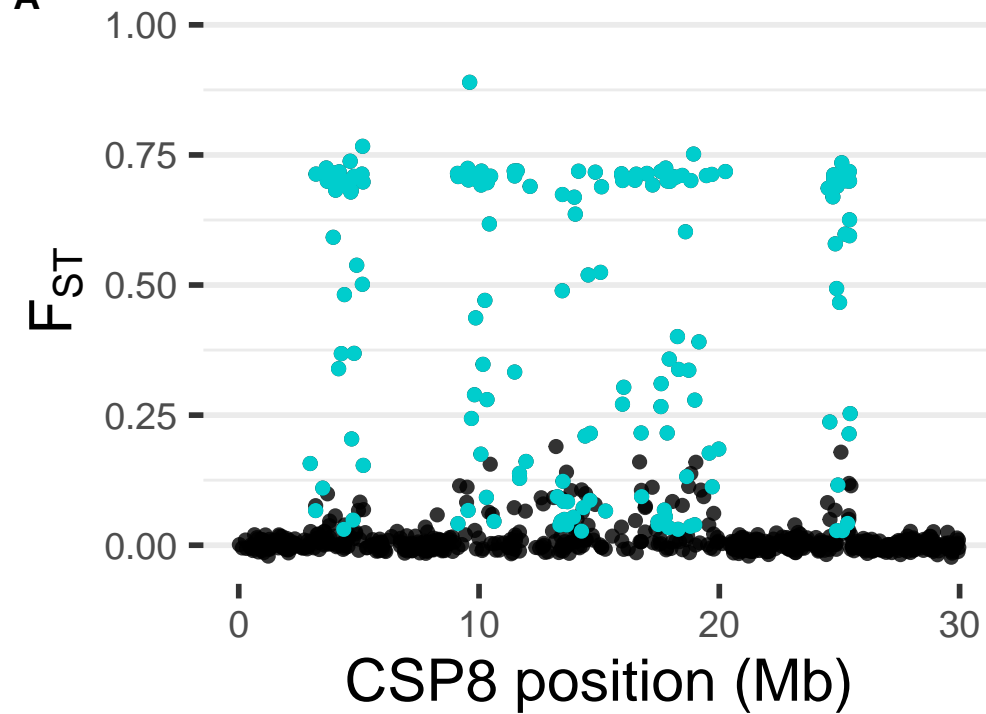**B**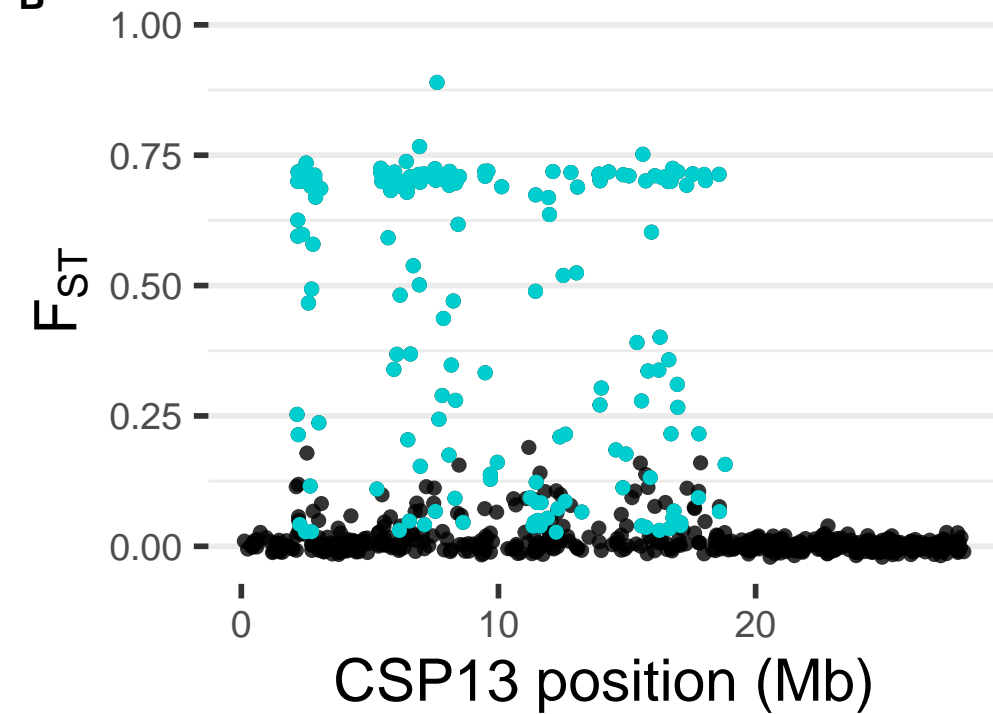**C**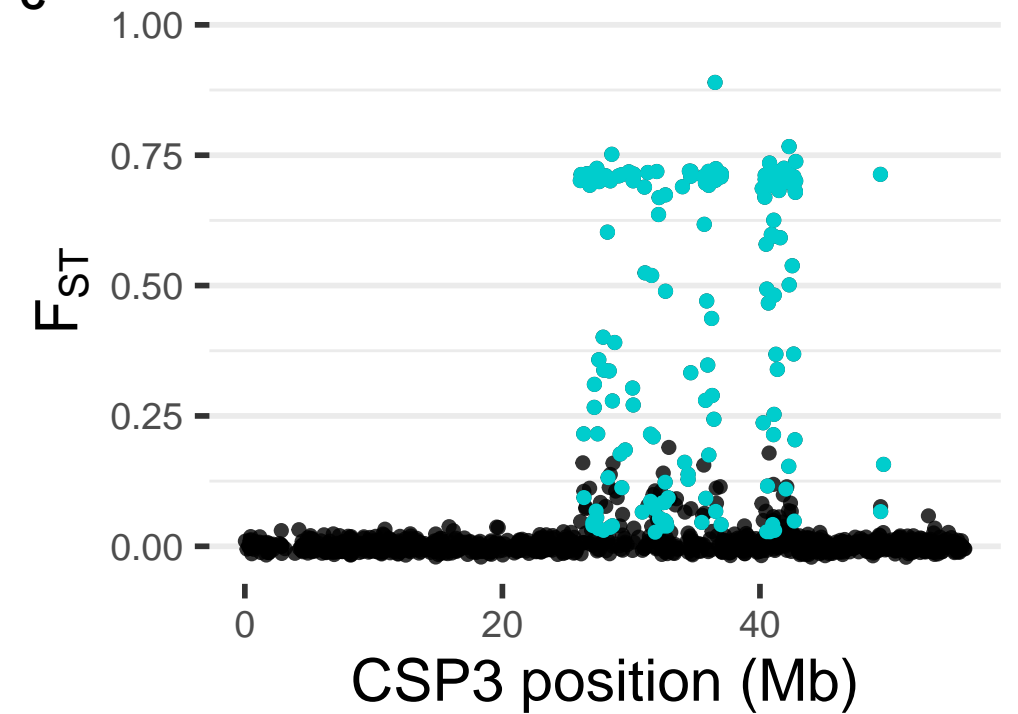

Supplement: Supplementary file 10 — Fig S10 [file EVA-13-2536-s010.pdf]

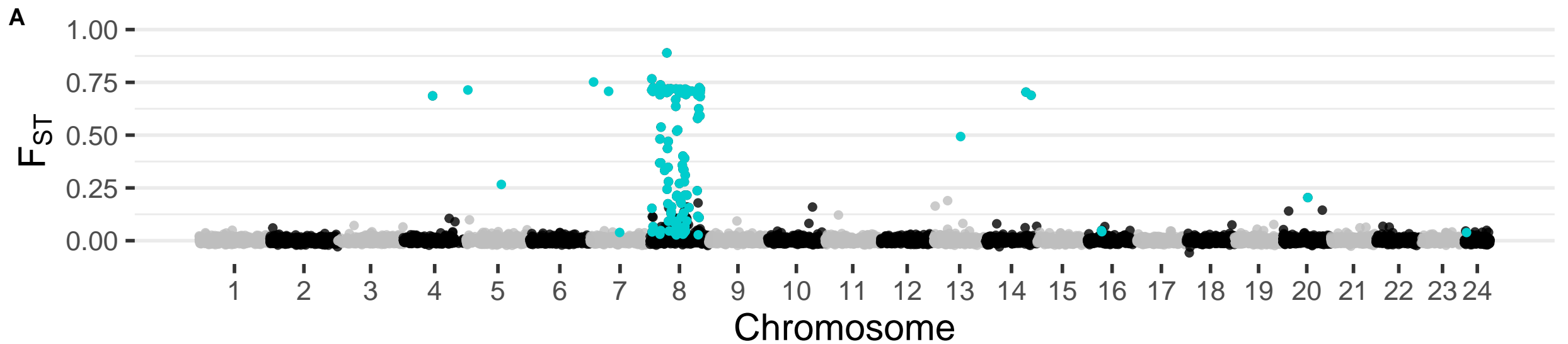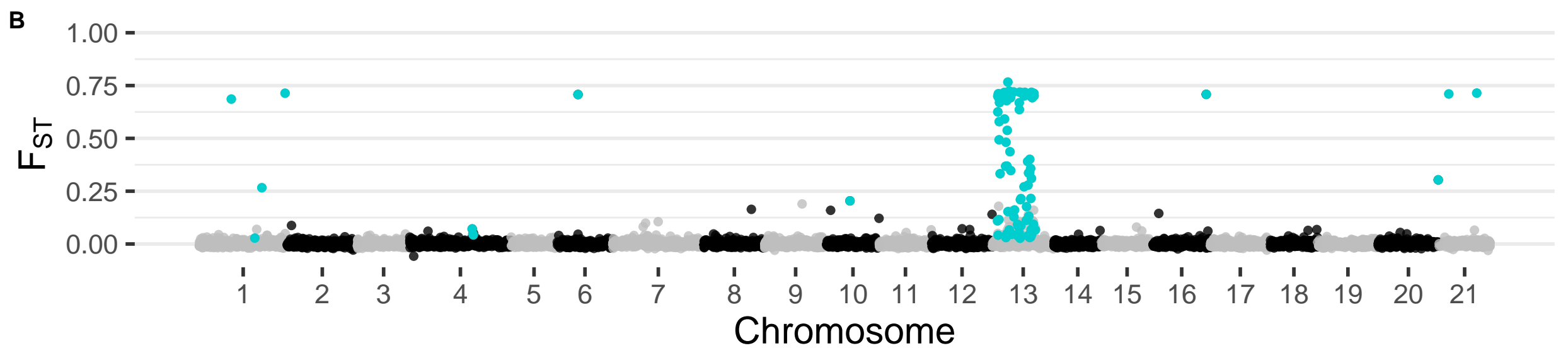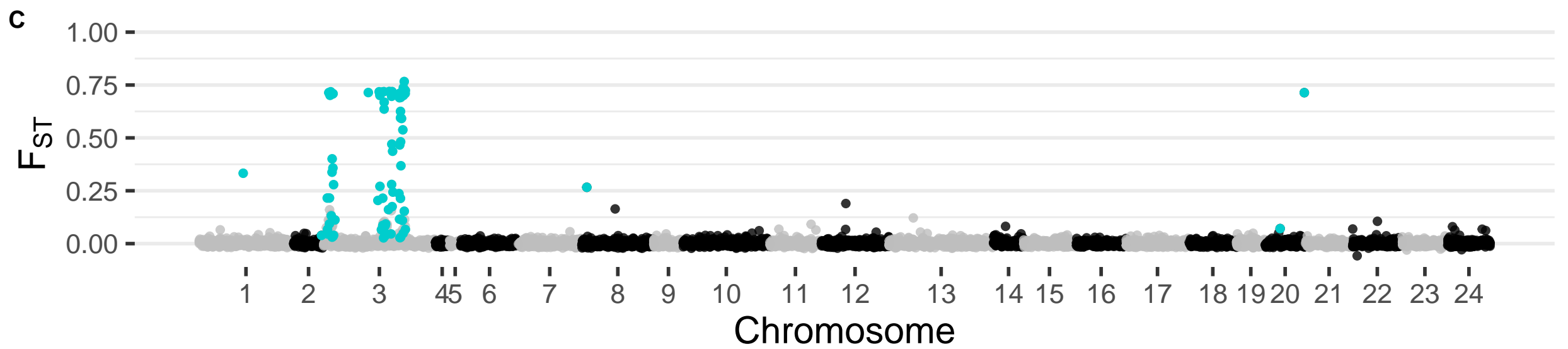

Supplement: Supplementary file 11 — Fig S11 [file EVA-13-2536-s011.pdf]

**A** $F_{ST}$ 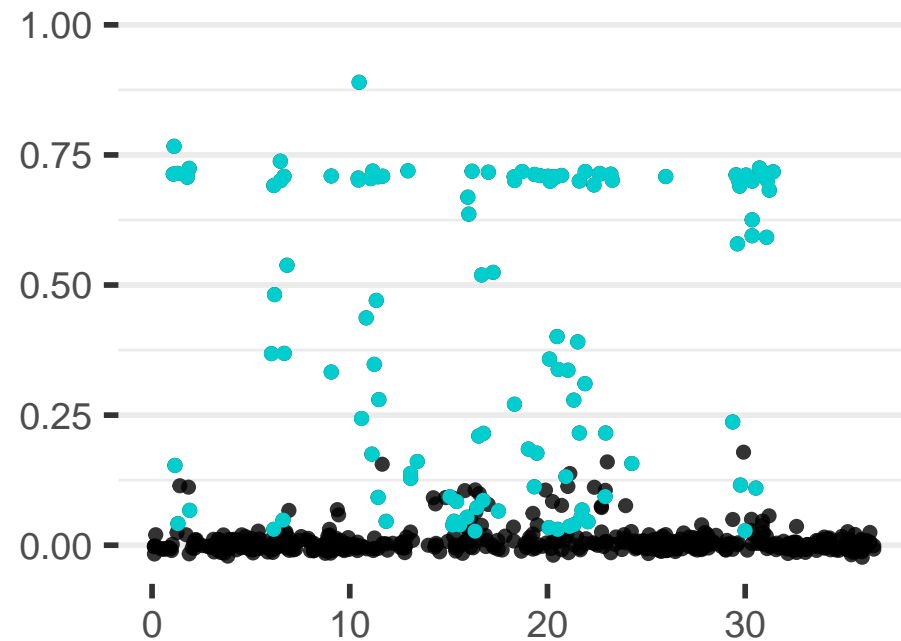

CHR8 position (Mb)

**B** $F_{ST}$ 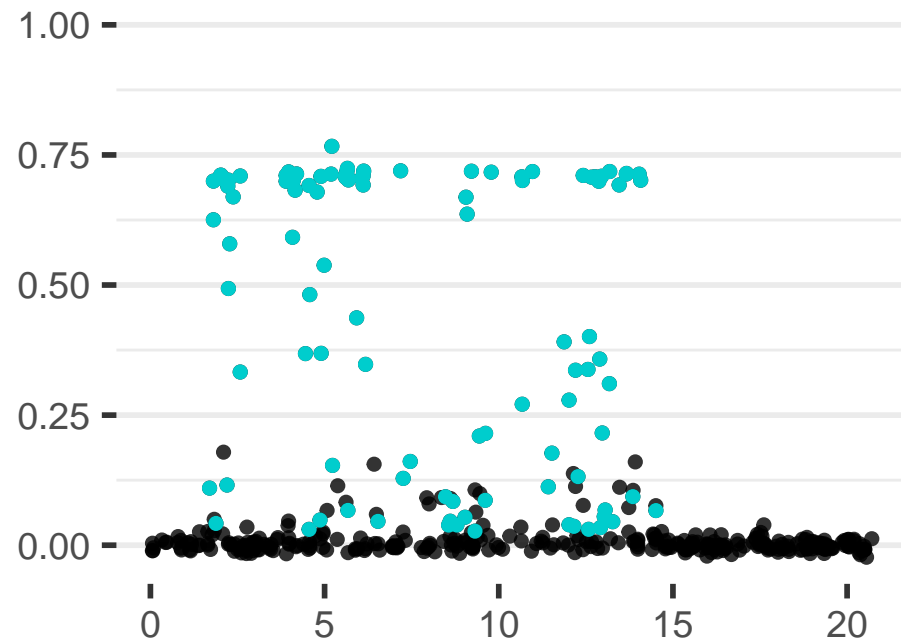

CHR13 position (Mb)

**C** $F_{ST}$ 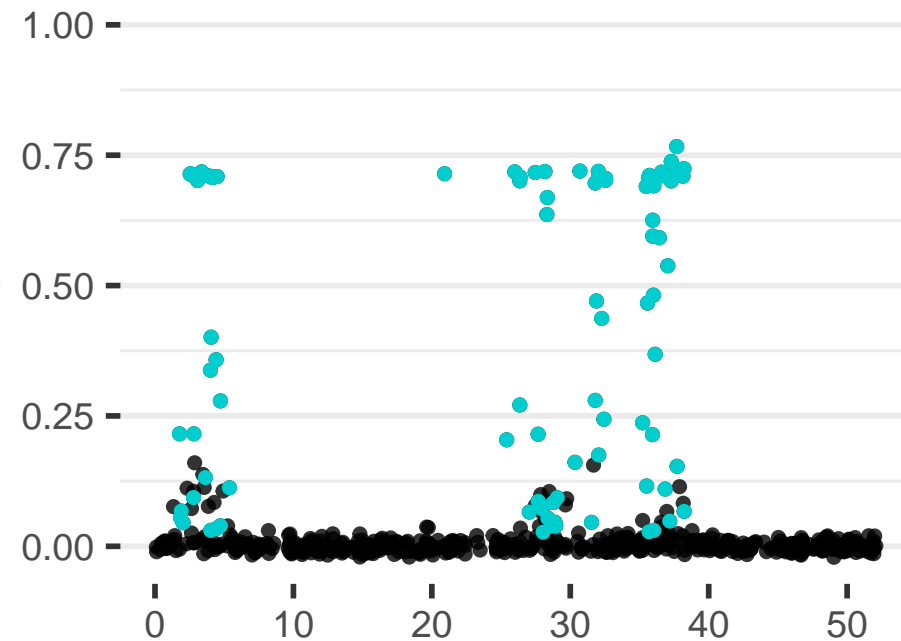

CHR3 position (Mb)

Supplement: Supplementary file 12 — Fig S12 [file EVA-13-2536-s012.pdf]

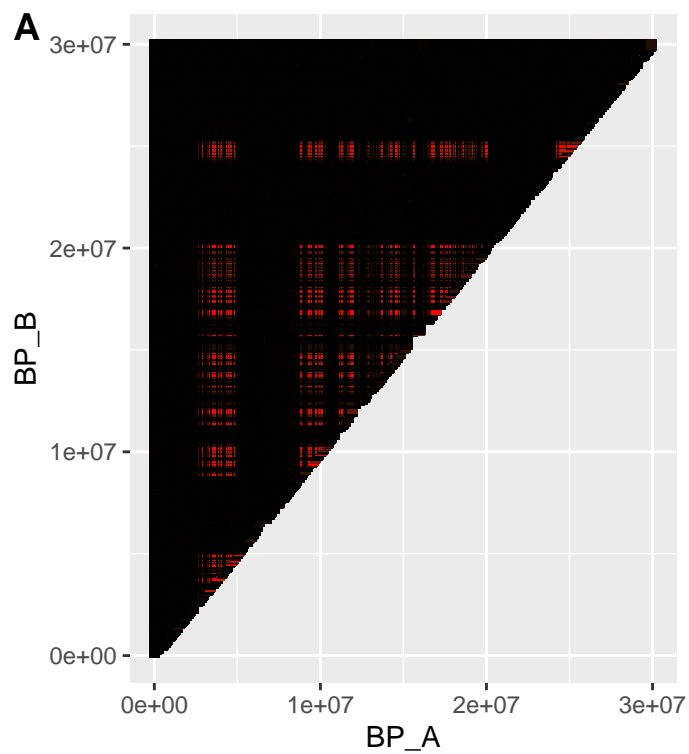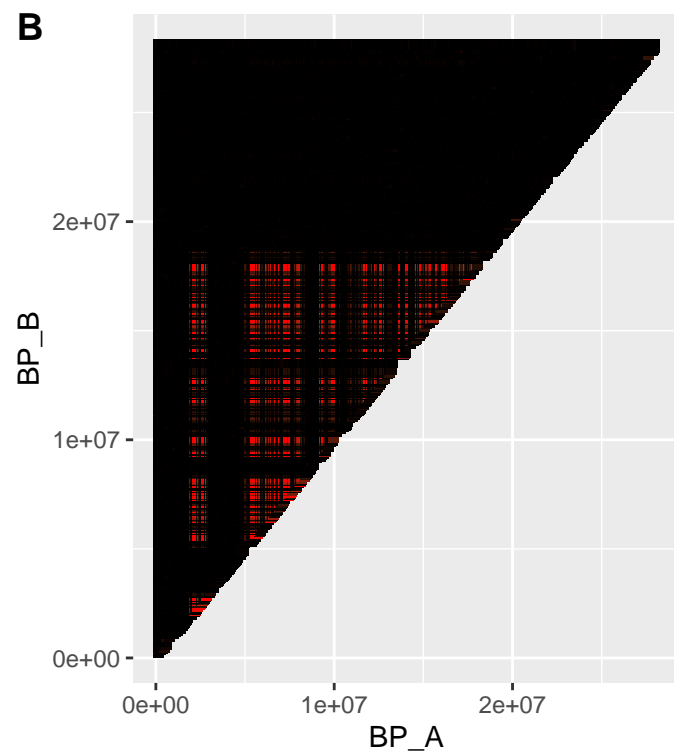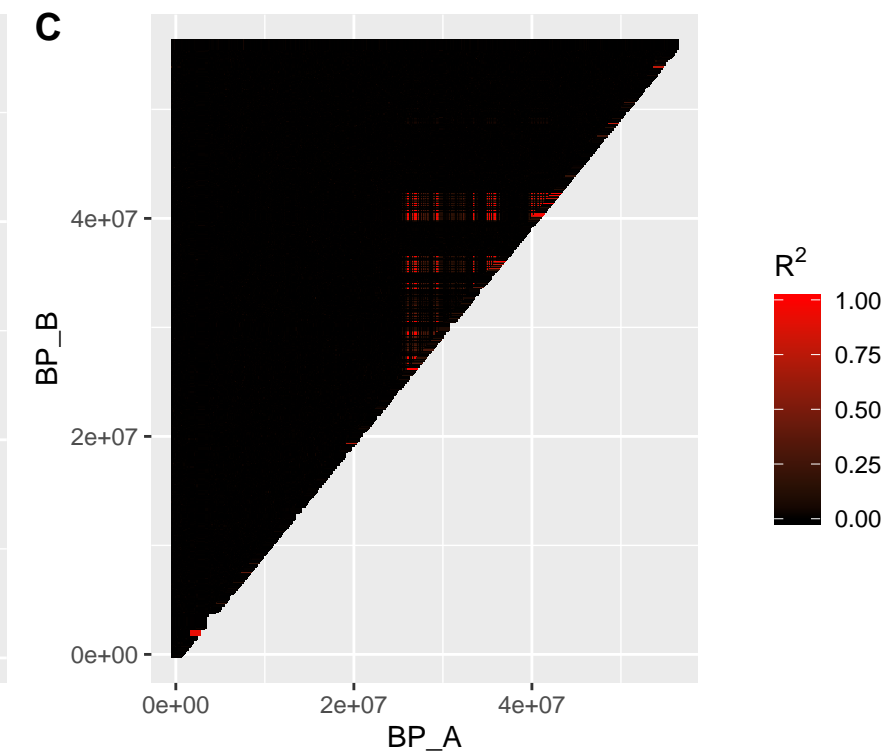

Supplement: Supplementary file 13 — Fig S13 [file EVA-13-2536-s013.pdf]

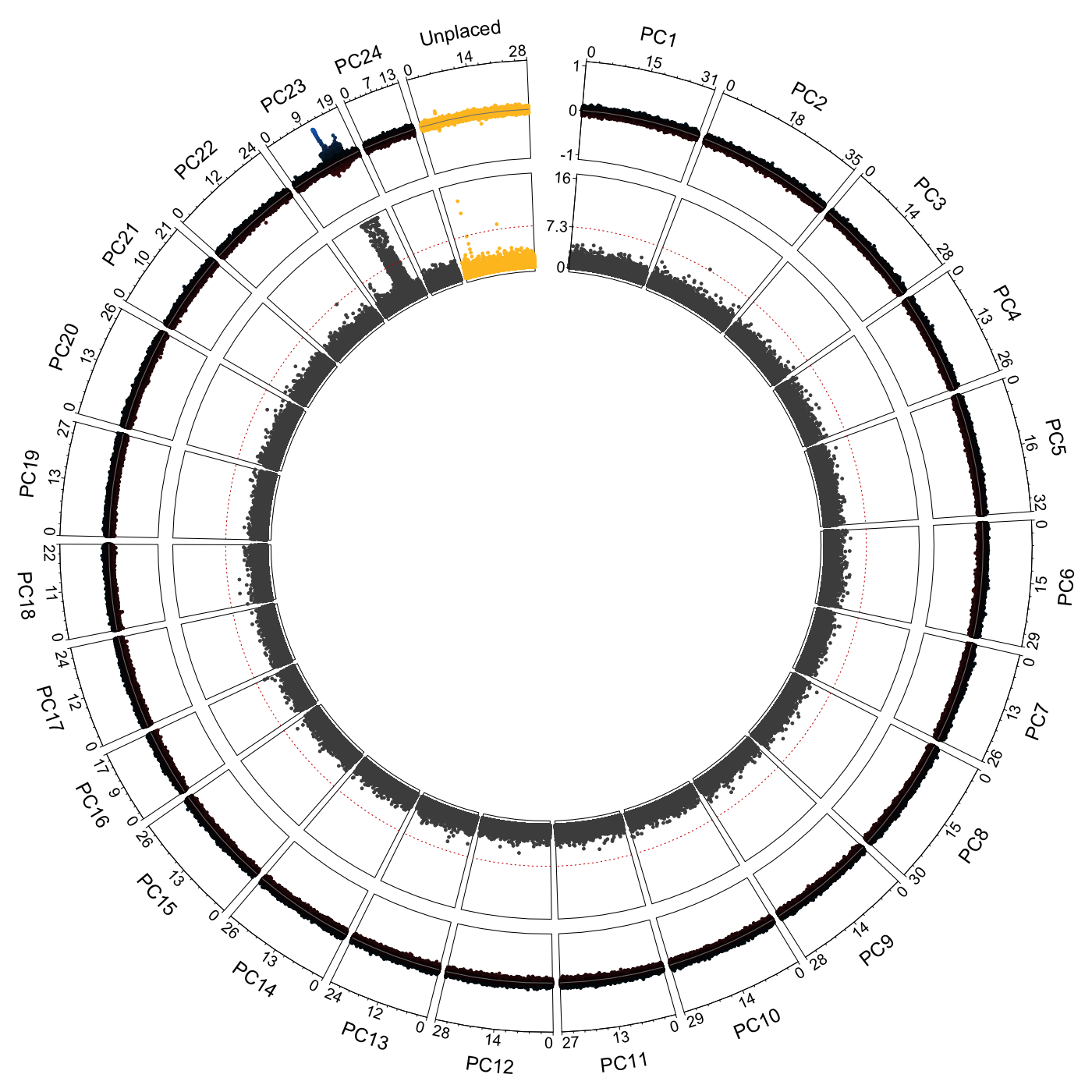

Supplement: Supplementary file 14 — Fig S14 [file EVA-13-2536-s014.png]
