# Supplementary material for: Strong population differentiation in lingcod (Ophiodon elongatus) is driven by a small portion of the genome
Source: Evol Appl. 2020 Jun 29;13(10):2536–54. doi: 10.1111/eva.13037 (PMC7691466; doi:10.1111/eva.13037)
Supplement: Supplementary file 16 — Table S2 [file EVA-13-2536-s016.docx]

**region site # site name n lat long N hap S hap NN NS SS**

GOA 1 South Montague 7 59.1815 -147.9763 1.000 0.000 1.000 0.000 0.000

GOA 2 East Kodiak 10 58.4054 -151.0168 1.000 0.000 1.000 0.000 0.000

GOA 3 West Chirikof 10 55.7728 -156.8757 1.000 0.000 1.000 0.000 0.000

AK 4 Nearshore Yakutat 41 59.5388 -139.9117 0.963 0.037 0.927 0.073 0.000

AK 5 ADFG stat area 375832 10 58.6167 -137.7500 1.000 0.000 1.000 0.000 0.000

AK 6 ADFG stat area 395800 16 58.3333 -139.1667 1.000 0.000 1.000 0.000 0.000

AK 7 ADFG stat area 385800 18 58.3333 -138.5833 1.000 0.000 1.000 0.000 0.000

AK 8 ADFG stat area 355702 8 57.1167 -136.0833 1.000 0.000 1.000 0.000 0.000

AK 9 Sitka 19 57.1614 -135.7697 1.000 0.000 1.000 0.000 0.000

AK 10 Beta Rock 18 55.6709 -133.7392 1.000 0.000 1.000 0.000 0.000

AK 11 Dall Island 17 55.1640 -133.3629 1.000 0.000 1.000 0.000 0.000

AK 12 Wolf Rocks 19 55.0157 -133.4981 1.000 0.000 1.000 0.000 0.000

CAN 13 Hecate Strait 7 53.6362 -131.0692 1.000 0.000 1.000 0.000 0.000

CAN 14 Queen Charlotte Sound 12 52.1842 -129.2015 1.000 0.000 1.000 0.000 0.000

CAN 15 Shellfish Section 14 48.9140 -125.4190 1.000 0.000 1.000 0.000 0.000

SS 16 San Juan Islands 11 48.4712 -123.0710 1.000 0.000 1.000 0.000 0.000

SS 17 Hood Canal 7 47.7155 -122.8790 1.000 0.000 1.000 0.000 0.000

SS 18 North Puget Sound 29 48.1415 -122.7057 1.000 0.000 1.000 0.000 0.000

SS 19 South Puget Sound 8 47.7041 -122.4821 0.938 0.063 0.875 0.125 0.000

WA 20 Cape Alava 17 48.1543 -124.7720 1.000 0.000 1.000 0.000 0.000

WA 21 Offshore La Push 16 48.0056 -125.3120 0.969 0.031 0.938 0.063 0.000

WA 22 Ocean Park 14 46.5353 -124.4240 1.000 0.000 1.000 0.000 0.000

OR 23 Cannon Beach 14 45.8400 -124.0219 1.000 0.000 1.000 0.000 0.000

OR 24 West Garibaldi 24 45.7167 -124.3524 0.958 0.042 0.917 0.083 0.000

OR 25 Stonewall Bank 27 44.5362 -124.4126 0.944 0.056 0.889 0.111 0.000

OR 26 Coos Bay 21 43.2535 -124.4637 0.952 0.048 0.905 0.095 0.000

OR 27 Mack Rock 16 42.2296 -124.4092 0.906 0.094 0.875 0.063 0.063

OR 28 Pt St George Reef 19 41.8230 -124.3624 0.842 0.158 0.737 0.211 0.053

N_CA 29 Reading Rock 9 41.3428 -124.1875 0.778 0.222 0.667 0.222 0.111

N_CA 30 Cape Mendocino 16 40.4731 -124.4932 0.813 0.188 0.688 0.250 0.063

N_CA 31 MacKerricher 9 39.4739 -123.8281 0.667 0.333 0.333 0.667 0.000

N_CA 32 Stewarts Pt 19 38.6116 -123.3797 0.605 0.395 0.368 0.474 0.158

N_CA 33 Offshore Pt Reyes 10 38.0606 -123.2999 0.300 0.700 0.100 0.400 0.500

C_CA 34 Farallon Islands 11 37.5042 -122.8582 0.182 0.818 0.091 0.182 0.727

C_CA 35 Big Sur 16 36.2082 -121.8235 0.156 0.844 0.000 0.313 0.688

C_CA 36 North Pt Conception 6 35.2333 -120.9204 0.083 0.917 0.000 0.167 0.833

S_CA 37 Santa Barbara Channel 10 34.2014 -119.6268 0.000 1.000 0.000 0.000 1.000

S_CA 38 Carrington Pt 14 34.0759 -120.0435 0.107 0.893 0.000 0.214 0.786

S_CA 39 Osbourne Bank 9 33.3636 -119.0547 0.000 1.000 0.000 0.000 1.000

S_CA 40 San Nicolas Island 18 33.3203 -119.4774 0.028 0.972 0.000 0.056 0.944

S_CA 41 San Diego 12 32.7735 -117.4687 0.000 1.000 0.000 0.000 1.000

BA 42 Colonet 3 30.7467 -116.5675 0.000 1.000 0.000 0.000 1.000
